# Dual role of the *Toxoplasma gondii* clathrin adaptor AP1 in the sorting of rhoptry and microneme proteins and in parasite division

**DOI:** 10.1371/journal.ppat.1006331

**Published:** 2017-04-21

**Authors:** Kannan Venugopal, Elisabeth Werkmeister, Nicolas Barois, Jean-Michel Saliou, Anais Poncet, Ludovic Huot, Fabien Sindikubwabo, Mohamed Ali Hakimi, Gordon Langsley, Frank Lafont, Sabrina Marion

**Affiliations:** 1 Centre d'Infection et d'Immunité de Lille, Université de Lille, Inserm U1019, CNRS UMR 8204, CHU Lille, Institut Pasteur de Lille, Lille, France; 2 IAB, Team Host-pathogen interactions & immunity to infection, Université Grenoble Alpes, Inserm U1209, CNRS UMR5309, Grenoble, France; 3 Laboratoire de Biologie Cellulaire Comparative des Apicomplexes, Faculté de Médicine, Université Paris Descartes—Sorbonne Paris Cité, France. Inserm U1016, CNRS UMR8104, Institut Cochin, Paris, France; Johns Hopkins School of Public Health, UNITED STATES

## Abstract

*Toxoplasma gondii* possesses a highly polarized secretory system, which efficiently assembles *de novo* micronemes and rhoptries during parasite replication. These apical secretory organelles release their contents into host cells promoting parasite invasion and survival. Using a CreLox-based inducible knock-out strategy and the ddFKBP over-expression system, we unraveled novel functions of the clathrin adaptor complex *Tg*AP1. First, our data indicate that AP1 in *T*. *gondii* likely functions as a conserved heterotetrameric complex composed of the four subunits γ, β, μ1, σ1 and interacts with known regulators of clathrin-mediated vesicular budding such as the unique ENTH-domain containing protein, which we named Epsin-like protein (*Tg*EpsL). Disruption of the μ1 subunit resulted in the mis-sorting of microneme proteins at the level of the Trans-Golgi-Network (TGN). Furthermore, we demonstrated that *Tg*AP1 regulates rhoptry biogenesis by activating rhoptry protein exit from the TGN, but also participates in the post-Golgi maturation process of preROP compartments into apically anchored club-shaped mature organelles. For this latter activity, our data indicate a specific functional relationship between *Tg*AP1 and the Rab5A-positive endosome-like compartment. In addition, we unraveled an original role for *Tg*AP1 in the regulation of parasite division. APμ1-depleted parasites undergo normal daughter cell budding and basal complex assembly but fail to segregate at the end of cytokinesis.

## Author summary

The phylum Apicomplexa comprises a large group of obligate intracellular parasites of wide human and agricultural significance. Most notable are *Plasmodium*, the causative agent of malaria, and *Toxoplasma gondii*, one of the most common human parasites, responsible for disease of the developing fetus and immune-compromised individuals. Apicomplexa are characterized by the presence of an apical complex consisting of secretory organelles named micronemes (MIC) and rhoptries (ROP). MIC and ROP proteins, released upon host cell recognition, are essential for host cell invasion and parasite survival. After invasion, these organelles are neo-synthesized at each parasite replication cycle. In our study, we demonstrate a crucial role for the *T*. *gondii* clathrin adaptor complex AP1 in the vesicular transport of neo-synthesized MIC and ROP proteins, thereby regulating mature apical organelle formation. In addition, we unravel an original role for *Tg*AP1 in the late stages of the parasite division process during daughter cell segregation. Therefore, our study provides new insights into key regulatory mechanisms of the vesicular trafficking system essential for host invasion and intracellular survival of *Toxoplasma gondii*.

## Introduction

Eukaryotic parasitic pathogens belonging to the phylum *Apicomplexa* are responsible for causing severe mortality in humans and great economic losses in livestock. *Toxoplasma gondii* (*T*. *gondii*) is of critical importance to pregnant women, as primary infections have the potential to cause neonatal malformations and even death of the developing foetus. In addition, the opportunistic nature of this obligate intracellular parasite can lead to the development of encephalitis in immunosuppressed individuals after reactivation of lifelong persistent cysts in the central nervous system [1]. As their name suggests *Apicomplexa* have a complex of unique apical secretory organelles called rhoptries, micronemes and dense granules that sequentially release their content enabling parasite invasion and intracellular survival. Microneme proteins (MIC) and Rhoptry Neck proteins (RON) are first secreted and trigger the formation of a transient structure, the moving junction (MJ) that anchors the parasite to the host cell and forms a ring through which the parasite penetrates [2] [3]. Rhoptry protein (ROP) contained in the bulb portion of these club-shaped organelles are immediately discharged after MJ formation and participate in the establishment of the intracellular parasitophorous vacuole (PV) in which the parasite intensively multiplies [4]. ROP proteins secreted into the host cell also play a crucial role in the manipulation of host innate immune responses to promote parasite survival [5]. Dense granule proteins (GRAs) are key parasite effectors exocytosed during parasite entry into the vacuolar space, where a certain sub-population contributes to the formation of a nano-tubulo-vesicular network called the intravacuolar network [6] [7]. This tubular network has been shown to be essential for nutrient import and regulation of parasite antigen exposure at the PV [8]. In addition, similar to ROP proteins, GRA proteins can be secreted beyond the PV membrane to actively modulate host gene expression and immune responses triggered upon infection [9].

The stripped-down and polarized version of the eukaryotic intracellular trafficking system has facilitated the use of *T*. *gondii* in studying the biogenesis of conserved organelles like the Golgi apparatus [10], and, more recently, of the apicomplexan-specific rhoptries, micronemes and dense granules [11]. These secretory organelles are formed *de novo* during each parasite replication cycle by budding and fusion of vesicles emerging from the ER and Golgi. Earlier studies have characterized sorting motifs within MIC and ROP proteins required for their trafficking from the Golgi towards their final destination [12] [13] [14] [15] [16] [17]. These studies led to the conclusion that protein processing and protein sorting were inter-dependent activities. For instance, the prodomain of soluble MIC3, MIC5 and M2AP proteins was shown to be essential for targeting the proteins to the micronemes [14] [15] [18]. Processing of ROP proteins takes place at a post-Golgi level and by contrast to MIC proteins, the presence of the pro-region of ROP1 was not a prerequisite for its targeting to the rhoptries [19]. More recently the trafficking routes taken by MIC and ROP proteins were delineated by examining the functions of some regulators of the endocytic compartments [11] [20] [21]. Key trafficking molecules were identified, such as the sortilin-like receptor (SORTLR) [22], the dynamin-related protein B (DrpB) [23] and the HOPS/ CORVET complex subunits Vps11, Vps18, Vps39, Mon1 and Vps9, recently described as the Guanine nucleotide Exchange Factor (GEF) of Rab5A [20] [24], all involved in the anterograde pathway regulating secretory organelle biogenesis. In addition, *Tg*Stx6, a parasite SNARE homolog of syntaxin 6 and the retromer protein Vps35, which are involved in the retrograde transport of molecules from the endosomal-like compartment (ELC) to the Golgi, were shown to be required for the biogenesis of dense granules [25] and rhoptries/micronemes [26], respectively. These recent findings suggest that *T*. *gondii* has functionally repurposed evolutionarily conserved regulators of the endosomal system to the secretory pathway to form secretory organelles [11] [20]. SORTLR was identified as the unique receptor transporting both, ROP and MIC proteins from the Golgi to the ELC [22]. Depletion of SORTLR led to parasites deprived of apical secretory organelles and ROP and MIC proteins were released into the vacuolar space, or the host cell cytoplasm via the default constitutive secretion pathway. However, so far, little is known about how neo-synthesized ROP and MIC proteins loaded on the unique receptor SORTLR are differentially sorted at the level of the Trans-Golgi-Network (TGN) to reach their distinct final destinations. ROP proteins and a sub-population of MIC proteins (MIC3/MIC8 complex) were shown to be transported via a Rab5A/C-dependent and Rab7-independent pathway, while MIC2/M2AP complex trafficking was Rab5A/C- and Rab7-independent [21]. However, depletion of Mon1, the putative GEF factor for Rab7, as well as depletion of the different HOPS complex subunits involved in Rab7 endosomal compartment biogenesis and integrity, was recently shown to impair rhoptry, microneme and dense granule formation, thus leading to contradictory conclusions concerning the role of Rab7 in secretory organelle formation [20]. Interestingly, the clathrin adaptor protein 1 complex (*Tg*AP1) was found associated with the C-terminal tail of SORTLR, suggesting that SORTLR-mediated transport of ROP and MIC proteins might occur via *Tg*AP1- and clathrin-dependent budding from the Golgi [22]. In eukaryotes, the AP1 complex has a highly conserved regulatory function in the transport of cargos at the level of the TGN and the early / sorting endosomal compartment [27] [28] [29]. Notably, AP1 regulates the targeting of resident hydrolases to lysosomes in mammals or to the digestive vacuole in plants [30] and yeast [27] [30]. AP1 also plays an essential role in the polarized sorting of vesicles from the TGN to the plasma membrane in epithelial cells [31] and in the secretion of plasma membrane and cell wall proteins in plants [30] [32]. In addition, AP1 has also been shown to have a conserved role in the regulation of the cell division process in lower and higher eukaryotes. For the latter, AP1 is crucial for the final step of daughter cell segregation by delivering Golgi-derived vesicles at the cleavage furrow of dividing cells or the developing cell plate in plants [33] [34] [35]. Finally, AP1 is also involved in the retrograde pathway from the early/sorting endosomes to the TGN [29] and in the retrieval of membrane and other factors from immature secretory granules to promote their maturation [36]. A recent phylogenic analysis of AP complexes in apicomplexans revealed that these parasites have undergone repeated secondary losses of adaptin complex genes [37]. While the four subunits of the AP1 complex were retained in all studied apicomplexan genomes, the entire AP3 complex was neither found in *Theileria*, nor in *Cryptosporidium parvum* and *Babesia bovis*. This study also indicated a possible degeneration of AP3 subunits in *Plasmodium*, while APμ2 was lost in *C*. *parvum*. In addition, like many other eukaryotes, the apicomplexans possess a single AP β1/2 subunit. Therefore, *T*. *gondii* appears as the unique apicomplexan parasite having conserved in its genome all the genes encoding for AP1, AP2, AP3 and AP4 complexes [37]. AP1 is composed of four subunits: two large subunits γ and β, a medium subunit μ, and a small subunit σ. Sorting motifs present in the cytoplasmic domain of cargo receptors are specifically recognized by the different sub-units of the complex. While γ and σ recognize the dileucine motif, β and μ bind to the tyrosine-based motif [27] [38]. *In T*. *gondii*, a mutagenesis analysis of the cytoplasmic domain (CD) of the transmembrane MIC2 protein has revealed that two conserved motifs are necessary and sufficient for targeting the protein to the micronemes [39]. One of these signals contains tyrosine residues, whereas the other one is composed of a stretch of acidic residues. These motifs are also present and conserved in the CD of MIC6 and are sufficient for microneme targeting [17]. These data suggested the existence of an AP-dependent mechanism for MIC protein sorting at the level of the TGN or ELC. Concerning ROP protein sorting mechanisms, a previous study indicated that ROP2 possesses a dileucine and a tyrosine-based motif located in the C-terminal part of the protein required for the export of ROP proteins from the TGN/ELC [40]. The authors of this study also demonstrated that these motifs were specifically recognized by the μ subunit of AP1 [40] [41]. Over-expressing APμ1 mutated at residues that bind the tyrosine-motif, led to accumulation of ROP2 in a post-Golgi multi-vesicular compartment resembling endosomes and immature rhoptries [41]. Similarly, perturbing the function of APμ1 by siRNA interference led to major defects in ROP biogenesis while, microneme and dense granule organelle biogenesis was not perturbed [41]. However, this model was challenged by the resolution of the ROP2 protein structure. This study revealed the absence of the predicted transmembrane domain and demonstrated that the association of ROP2 with the parasitophorous vacuole membrane is mediated by an amphipathic peptide enclosed in the N-terminal domain [42]. Of note, SORTLR that associates with ROP and MIC proteins also possesses a dileucine motif in its cytoplasmic tail, suggesting an additional AP-dependent sorting mechanism for ROP and MIC proteins [22]. In *P*. *falciparum*, AP1 localizes at the Golgi/ER compartment and in rhoptries at the schizont stages. The APμ1 subunit was found associated with the rhoptry-associated protein 1 (RAP1) suggesting a role in rhoptry protein trafficking [43].

In the present study, we demonstrated that *Tg*AP1 regulates both, rhoptry and microneme formation but not dense granule biogenesis. In addition, despite a significant difference in the cell division process of *T*. *gondii* compared to other eukaryotes, our study revealed a conserved role for *Tg*AP1 in the late stages of cytokinesis, by regulating the final step of daughter cell segregation.

## Results

### *Tg*APμ1 localizes at the Trans-Golgi-Network and on secretory vesicles

**Fig 1:**
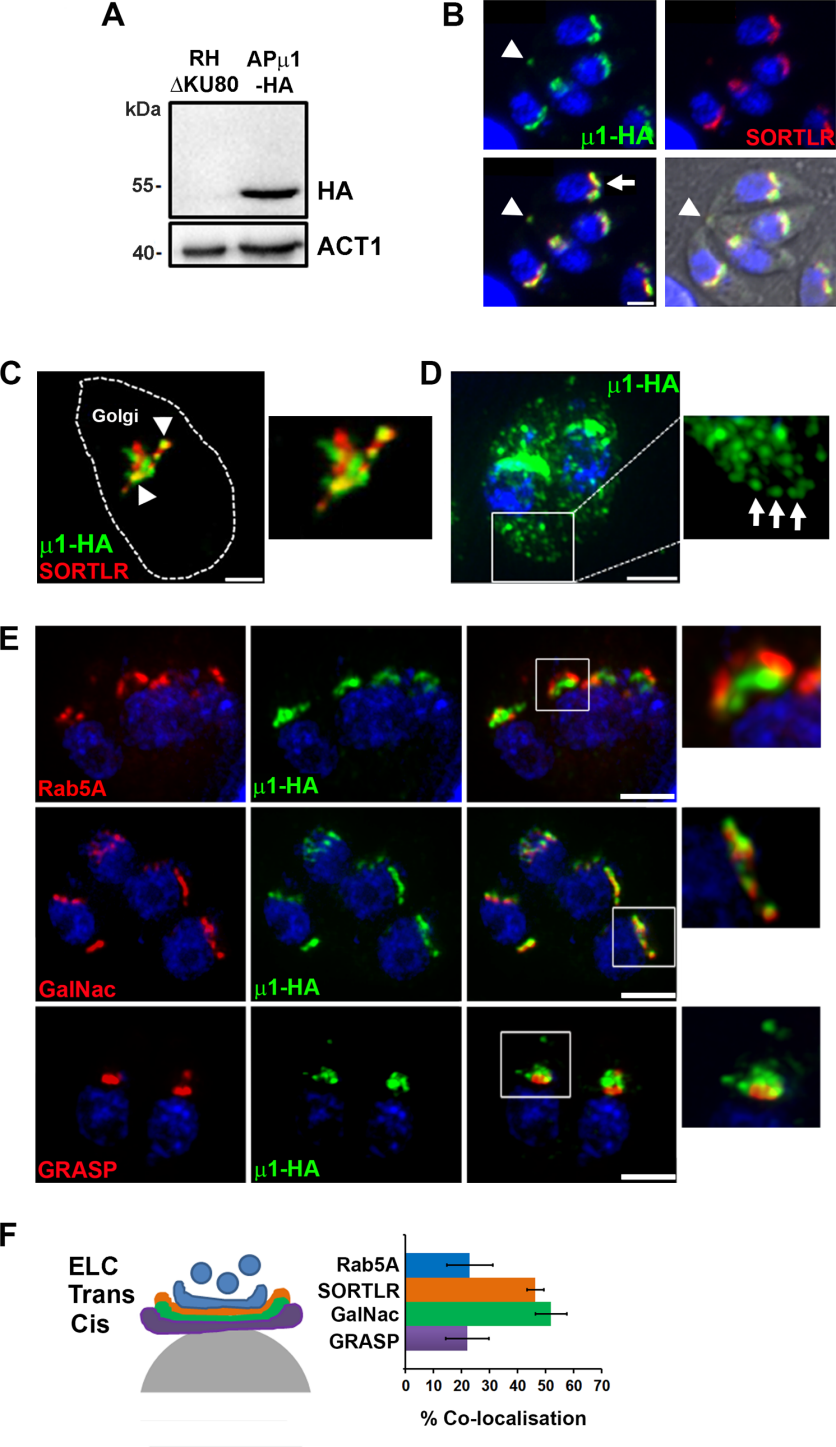
APμ1 localizes at the Trans-Golgi-Network and on secretory vesicles. **A-** Western Blot image showing the expression of the endogenous HA-tagged μ1 subunit at the expected size of 49 kDa in knock-in parasites (RHΔKU80: parental strain). Actin (ACT1) was used as a loading control. **B-** Confocal microscopy images showing the localization of μ1-HA (green) with SORTLR (red) at the Golgi region (arrow). Nuclei are shown by DNA staining (blue). Note the very discrete signal of μ1-HA (green) at a localization corresponding to the residual body (arrowhead). Bar: 2 μm. **C-** SIM image showing the partial co-localization (indicated by arrowheads) of μ1-HA (green) with SORTLR (red) in sub-regions of the Golgi apparatus. Bar: 1 μm. **D**- SIM image showing the localization of μ1-HA (green) in vesicles spread throughout the parasite cytoplasm and also present in proximity to the plasma membrane (arrows in the inset). Bar: 2 μm. **E-** The co-localization of μ1-HA (green) with markers of the endosomal compartment (Rab5A-YFP), trans-Golgi (GalNAc-GFP) and cis-Golgi (GRASP-RFP) (all shown in red) was examined by SIM microscopy. For each marker, a zoom of the Golgi region is shown (insets). Bars: 2 μm. **F-** Scheme (left) illustrating the localization of the different markers associated with the endosomal-like compartment (ELC), the trans-Golgi (Trans) and the cis-Golgi (Cis). The histogram (right) indicates the percentage of co-localization between μ1-HA and Rab5A-YFP, SORTLR, GalNAc-GFP and GRASP-RFP, in at least 30 parasites that were analyzed for each condition. μ1-HA displays the strongest co-localization with the TGN markers SORTLR and GalNAc-GFP.

In order to define the subcellular localization of the *Tg*AP1 complex, we generated knock-in (KI) parasites expressing the μ1 subunit (TGGT1_289770) fused to a HA tag at its C-terminus. Western blot analysis confirmed the expression of the tagged protein at the expected size (Fig 1A). A clonal parasite line was isolated and APμ1 localization was analyzed by an immunofluorescence assay (IFA) using confocal (Fig 1B) and super-resolution microscopy SIM (structured illumination microscopy)(Fig 1C and 1D). As expected for the AP1 complex, a localization at the Golgi area was observed and confirmed by co-localization with the TGN marker SORTLR (Fig 1B and 1C). In addition to the TGN, a faint but specific APμ1 signal was systematically detected in the parasite cytoplasm by confocal microscopy. After saturating the stronger Golgi-associated signal, we clearly identified this weaker signal as APμ1-positive vesicles spread throughout the cell cytoplasm and close to the cell periphery (Fig 1D, arrows). This pattern of distribution strongly suggested that *Tg*AP1 is involved in additional trafficking pathways apart from the ones involved in MIC and ROP protein transport, in particular, in vesicle delivery to the plasma membrane or the inner membrane complex (IMC).

SIM acquisition and Imaris software analysis indicated a 46.4 ± 3.8% co-localization between APμ1 and SORTLR (Fig 1C and 1F) at the TGN. In agreement, APμ1 shows a similar high percentage of co-localization (52.0 ± 5.6%) with another TGN marker, GalNAc, in contrast to the cis-Golgi marker GRASP (22.0 ± 7.7%) (Fig 1E and 1F). Using a KI line expressing Rab5A-YFP under the endogenous promotor, the Rab5A-positive endosome-like compartment (ELC) was mostly detected as vesicles emerging posteriorly from the APμ1- and SORTLR-positive TGN (Fig 1E, upper panel, Fig 1F and S1 and S2 Movies). In agreement, a weaker co-localization of APμ1 with Rab5A was quantified (22.9 ± 8.2%) compared to the TGN markers SORTLR and GalNac (Fig 1E and 1F). However, we noticed that the Rab5A-positive ELC and the TGN appeared physically connected, in particular during Golgi duplication at the G1/S transition phase of the cell cycle (S1A Fig). This notion was also supported by the observation that Brefeldin A (BFA) treatment, which disperses the Golgi apparatus by inhibiting the activity of the ARF1 GTPase required for COPII-mediated vesicular transport, led to the partial dispersion of the Rab5A-positive compartment similar to what was observed for the AP1-positive TGN (S1B Fig). As previously suggested [19], these results indicate that *T*. *gondii* possesses an unusal ELC, which is physically and likely functionally connected to the TGN, resembling the early endosomal/TGN hybrid compartment of plants [44].

### *Tg*APμ1 belongs to a conserved tetrameric complex and interacts with the epsin-like protein

**Fig 2:**
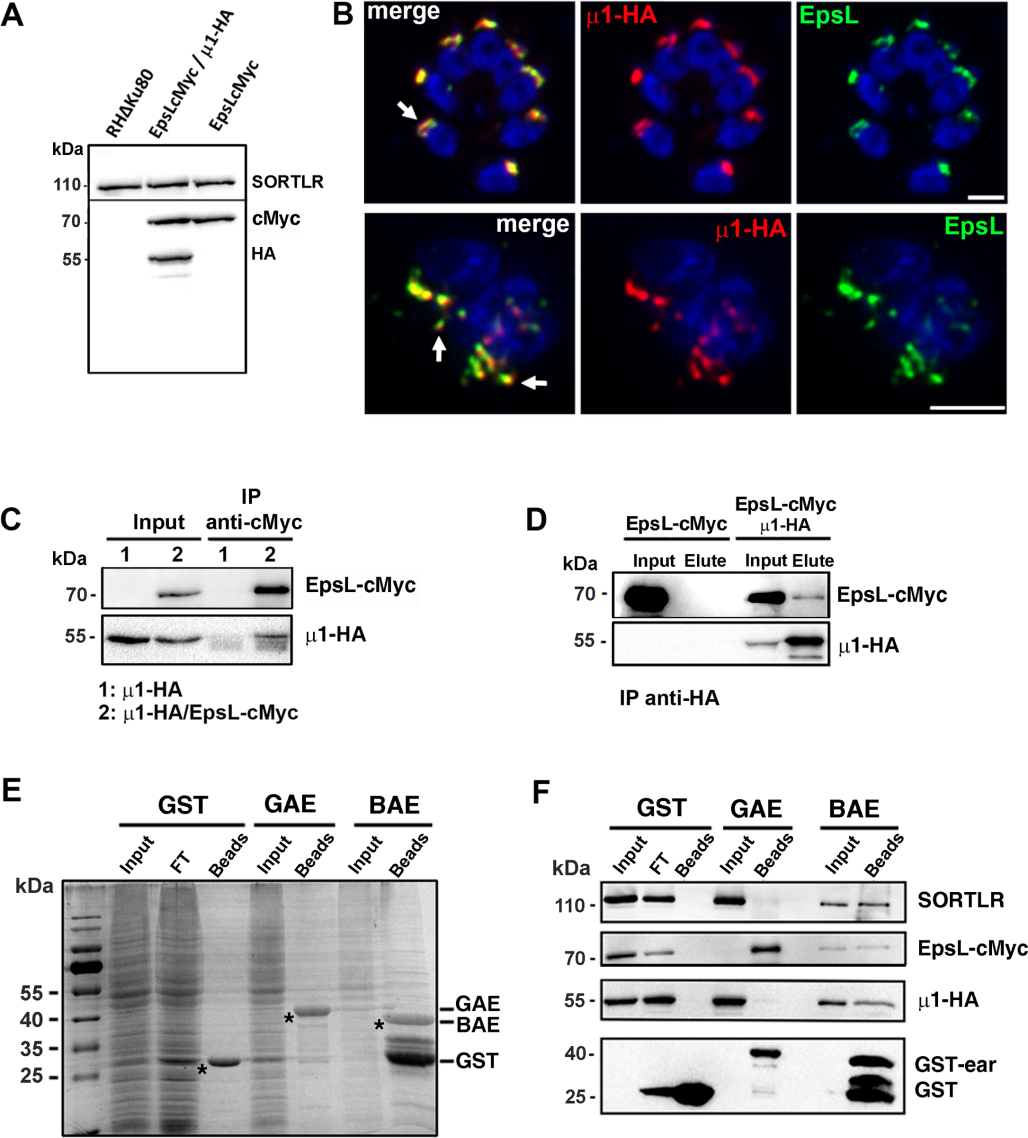
The unique ENTH-domain containing protein of *T*. *gondii* is a key partner of APμ1. A- Western blot showing the expression of the cMyc-tagged EpsL protein at the expected size of 66 kDa in single KI parasites (lane 2), and together with μ1-HA (49 kDa) in double knock-in parasites (lane 3). The parental strain (RHΔKU80) is shown in lane 1. SORTLR was used as a loading control. B- Images illustrating the co-localization of EpsL-cMyc (green) with μ1-HA (red) (arrows), acquired with confocal microscopy (upper panel) or SIM (lower panel). Bars: 2μm. C- Co-immunoprecipitation of μ1-HA with EpsL-cMyc in double KI parasites (lanes 2) using anti-cMyc antibodies. No binding of the μ1-HA protein on anti-cMyc coated beads was detected in single KI μ1-HA expressing parasites (lane 1). D- Reverse co-immunoprecipitation of EpsL-cMyc with μ1-HA in double KI parasites using anti-HA antibodies. No binding of EpsL-cMyc protein with anti-HA antibody coated beads was detected in the single KI EpsL-cMyc expressing parasites. E, F- A GST-pull down experiment with the GST-tagged gamma appendage ear (GAE) and GST-tagged beta appendage ear (BAE) domains of *Tg*AP1 was performed in presence of a total lysate from EpsL-cMyc/ *μ*1-HA double KI parasites. E: SDS-PAGE gel stained with coomassie blue showing that a similar quantity of GST, GST-GAE and GST-BAE (asterisks) was bound on the gluthation beads used in the assay shown in F. F: WB analysis demonstrated the preferential binding of EpsL-cMyc to the ear domain of the γ sub-unit (GAE). A weak interaction of SORTLR and the μ1 sub-unit with the BAE domain was also detected. GST alone was used as a control. FT: Flow-Through.

To assess, whether APμ1 is a component of the highly conserved tetrameric complex composed of the three other subunits σ1, β and γ, we performed an immunoprecipitation (IP) assay using anti-HA antibodies on APμ1-HA KI parasite lysate followed by mass spectrometry analysis. APμ1 was reproducibly found associated with the three other subunits (Table 1, S2 Fig) confirming the formation of a conventional tetrameric complex described in other eukaryotes. This finding was also supported by the localization of the σ1 sub-unit at the SORTLR-positive TGN in KI parasites expressing the fusion protein σ1-HA under the native promotor (S3A and S3B Fig). In addition, the unique ENTH domain-containing protein encoded in the *T*. *gondii* genome, which we named Epsin-like protein (*Tg*EpsL) was identified, however with only one unique peptide (Table 1, S2 Fig). We decided to further investigate the possible interaction between *Tg*AP1 and *Tg*EpsL because epsin proteins are very well known AP2 and AP1 binding proteins involved in the activation of clathrin-mediated vesicular budding at the plasma membrane and at the TGN, respectively. Epsins bind to phospholipids via their ENTH domain and regulate clathrin coat formation by inducing curvature of the lipid bilayers [38]. Sequence analysis indicated that *Tg*EpsL contains conserved clathrin and phosphoinositide binding sites, such as recently described elsewhere [45] (S3C Fig). However, besides the ENTH domain, no similarities were found between *Tg*EpsL and other epsins (S3C Fig), suggesting a specificity of binding partners and regulatory mechanisms of the protein activity in *T*. *gondii*. First, we generated single KI parasites expressing EpsL-cMyc under its natural promotor as well as double KI parasites expressing both, EpsL-cMyc and APμ1-HA proteins (Fig 2A). IFA analysis by confocal and SIM microscopy confirmed the co-localization of *Tg*EpsL with *Tg*AP1 at the TGN (Fig 2B). To verify the interaction between *Tg*AP1 and *Tg*EpsL, an IP was performed on double KI EpsL-cMyc / APμ1-HA expressing parasites, using either anti-cMyc or anti-HA antibodies. Western blot analysis confirmed the interaction between APμ1-HA and EpsL-cMyc in both IP assays (Fig 2C and 2D). In agreement with this result, IP of EpsL-cMyc followed by mass spectrometry identified the β, γ and μ1 subunits of the AP1 complex and the small GTPase ARF1 as the main proteins associated with *Tg*EpsL (Table 2, S4 Fig), thereby confirming the result obtained by western blot. Importantly, no subunit of the AP2 complex was identified, suggesting that *Tg*EpsL might not function in AP2-mediated endocytosis. In other eukaryotes, the AP1 complex interacts with epsinR via the exposed GAE (“Gamma Appendage Ear”) domain of the γ subunit [46] [47]. Sequence alignment analysis showed a strong conservation of *T*. *gondii* GAE and BAE (“Beta Appendage Ear”) domains with the corresponding domains of the AP1 β and γ subunits from other species (S5 Fig, S1 Table). Therefore, the GAE and BAE domains of *Tg*AP1, fused to GST were produced (Fig 2E). GST pull-down experiments in presence of a total extract of EpsL-cMyc/ APμ1-HA double KI parasites indicated that the GAE domain is sufficient to pull-down the *Tg*EpsL protein, while no binding of the μ1 subunit was monitored (Fig 2F). In contrast, a weak interaction was detected between *Tg*EpsL and the BAE domain (Fig 2F), suggesting a preferential association of *Tg*EpsL with the γ sub-unit, as a similar quantity of the two domains was used in the assay (Fig 2E). Interestingly, we also found that the BAE domain pulled down the μ1 subunit. As no direct interaction between this domain and the μ1 subunit has been described in other eukaryotes, it was likely that the BAE domain could interact with a complex of proteins that includes μ1 and other *Tg*AP1 binding proteins. In agreement with this finding, SORTLR that directly interacts with the μ1 subunit, was also found in the pull down eluate of the β-ear but not in the eluate of the γ-ear (Fig 2F).

**Table 1:** List of proteins identified by mass spectrometry following the IP of μ1-HA proteins in KI parasites expressing μ1-HA under the endogenous promotor. The detailed list is included in S2 Fig. The parental strain RHΔKU80 was used as a control for non-specific binding to the anti-HA antibody-coated beads. The table indicates the number of “unique peptides / spectra” for each identified protein in two biological independent assays (IP1 and IP2).

			Total number of unique peptides / spectra	
Protein name	Accession numbers	Molecular weight (Da)	IP-1	IP-2
			μ1-HA	μ1-HA
mu1 adaptin	TGGT1_289770	48 918	11/55	8/16
gamma adaptin	TGGT1_313670	107 036	20/53	24/77
beta adaptin	TGGT1_240870	101 920	18/47	19/76
sigma1 adaptin	TGGT1_270370	19 679	2/4	1/2
clathrin heavy chain	TGGT1_290950	194 480	4/6	1/1
ENTH-domain containing protein	TGGT1_214180	65 903	1/1	1/1

**Table 2:** List of proteins identified by mass spectrometry following the IP of EpsL-cMyc protein in double KI parasites expressing EpsL-cMyc and μ1-HA proteins. The detailed list is included in S4 Fig. The single KI parasites expressing μ1-HA was used as a control for non-specific binding to the anti-cMyc antibody-coated beads. The table indicates the number of “unique peptides / spectra” for each identified protein.

Protein name	Accession numbers	Molecular weight (Da)	Total number of unique peptides / spectra	
			Control:	IP:
			μ1-HA	EpsL-cmyc/μ1-HA
ENTH domain-containing protein	TGGT1_214180	65 903	3/4	15/76
beta adaptin	TGGT1_240870	101 920	/	19/28
gamma 1 adaptin	TGGT1_313670	107 036	2/3	17/33
mu1 adaptin	TGGT1_289770	48 918	/	9/11
ADP ribosylation factor ARF1	TGGT1_276140	61 745	2/2	6/12

Together these data indicate that the AP1 complex in *T*. *gondii* is conserved at the molecular level and likely functions similar to its mammalian homologue as a heterotetrameric complex regulating epsin-mediated vesicular transport of parasite proteins.

### *Tg*AP1 regulates microneme formation

**Fig 3:**
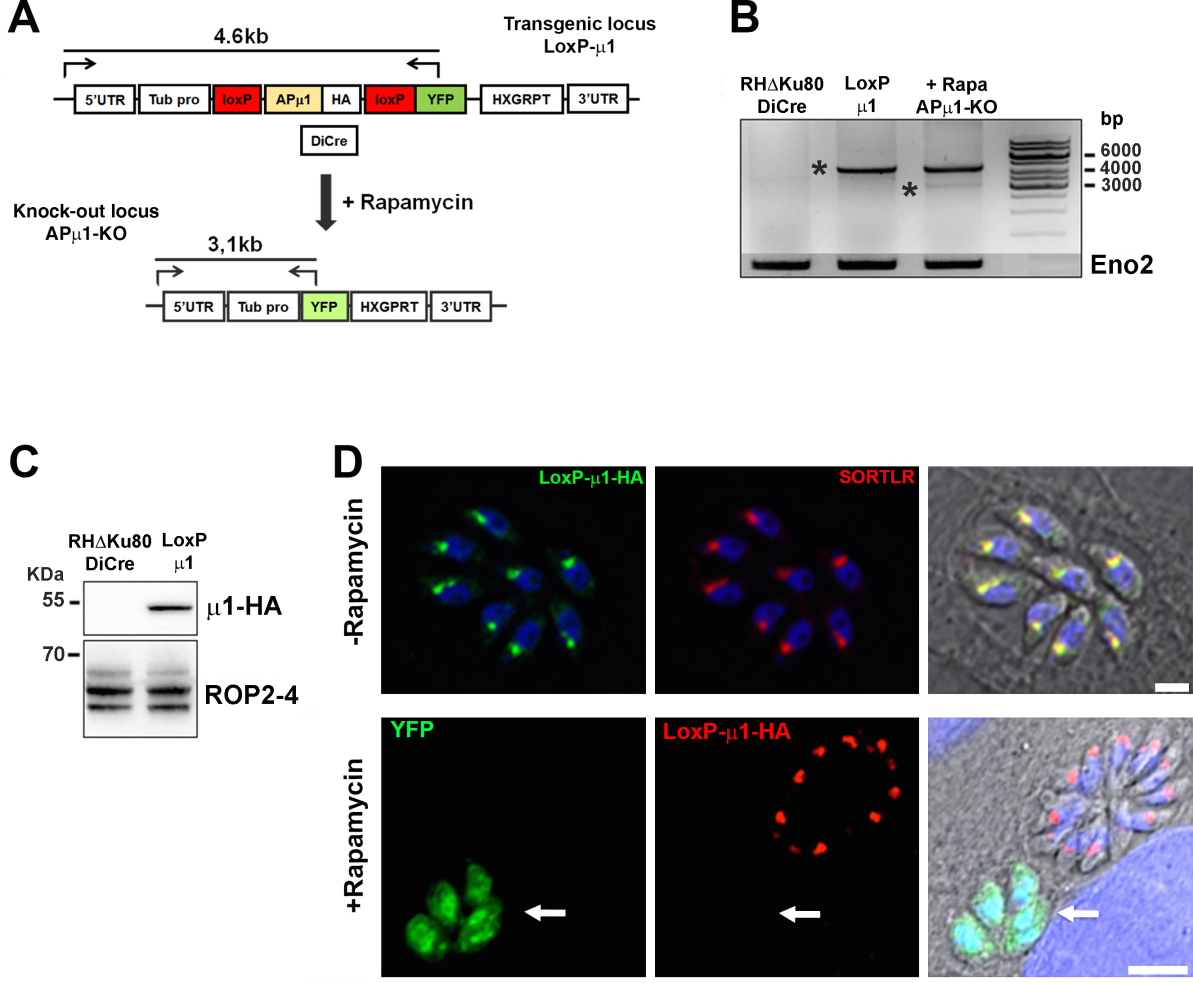
CreLox-based strategy used to deplete APμ1. **A-** Scheme depicting the cloning strategy used to replace the endogenous μ1 locus by the LoxP-μ1-HA-LoxP insert. Upon rapamycin induction, the DiCre recombinase excised the LoxP flanked locus leading to YFP expression. The positions of the primers used to verify the integration of the insert into the genome and its excision upon rapamycin incubation, are indicated. **B-** PCR confirming the integration of the LoxP-μ1-HA-LoxP insert at the endogenous APμ1 locus resulting in the amplification of a band at 4.6 kb (asterisk). Rapamycin induction resulted in the amplification of a lower and weak band at 3.1 kb (asterisk) corresponding to the low percentage of APμ1-KO parasites. The primers used for the PCR are depicted in A-. Amplification of the enolase 2 (Eno2) gene was used as a control. **C-** WB showing the expression of integrated μ1-HA protein at the expected size in a clonal population (DiCre RHΔKU80: parental strain). ROP 2–4 was used as a loading control. **D- Upper panel:** Confocal microscopy images showing the localization of μ1-HA (green) together with SORTLR (red) at the TGN, after integration of the sequence flanked by the LoxP sites in DiCre RHΔKU80 parasites. Nuclei are shown by DNA staining (blue). Bar: 2μm. **Lower panel:** Confocal microscopy images showing the absence of μ1-HA signal (red) in YFP positive parasites (arrow) upon rapamycin treatment. Bars: 5μm.

To investigate *Tg*AP1 functions, inducible knock-out (KO) parasites lacking the μ1 subunit were generated using the CreLox strategy [48], where excision of the endogenous locus and subsequent expression of the YFP protein is triggered upon addition of rapamycin (Fig 3A). Integration of the LoxP-μ1HA-LoxP cassette at the endogenous locus was validated by PCR (Fig 3B) and expression of the corresponding protein by Western Blot (Fig 3C). By IFA, integration of the LoxP-μ1HA-LoxP cassette led to the expression of a tagged protein that co-localized with SORTLR at the TGN (Fig 3D, upper panel). Incubation of transgenic parasites with rapamycin triggered APμ1 depletion and YFP expression, but only in 12.0±4% of the whole population (Fig 3B and 3D, lower panel). Of note, increasing rapamycin concentration did not lead to a higher yield of KO parasites. Longer rapamycin induction periods improved the rate of YFP-positive parasites but led to random excision events over the successive cell cycles making it difficult to analyze the heterogenous phenotypes associated with AP1 depletion. Thus, we decided to induce APμ1-KO parasites for 6 hours after invasion in order to trigger μ1 gene exision during the first division cyle.

**Fig 4:**
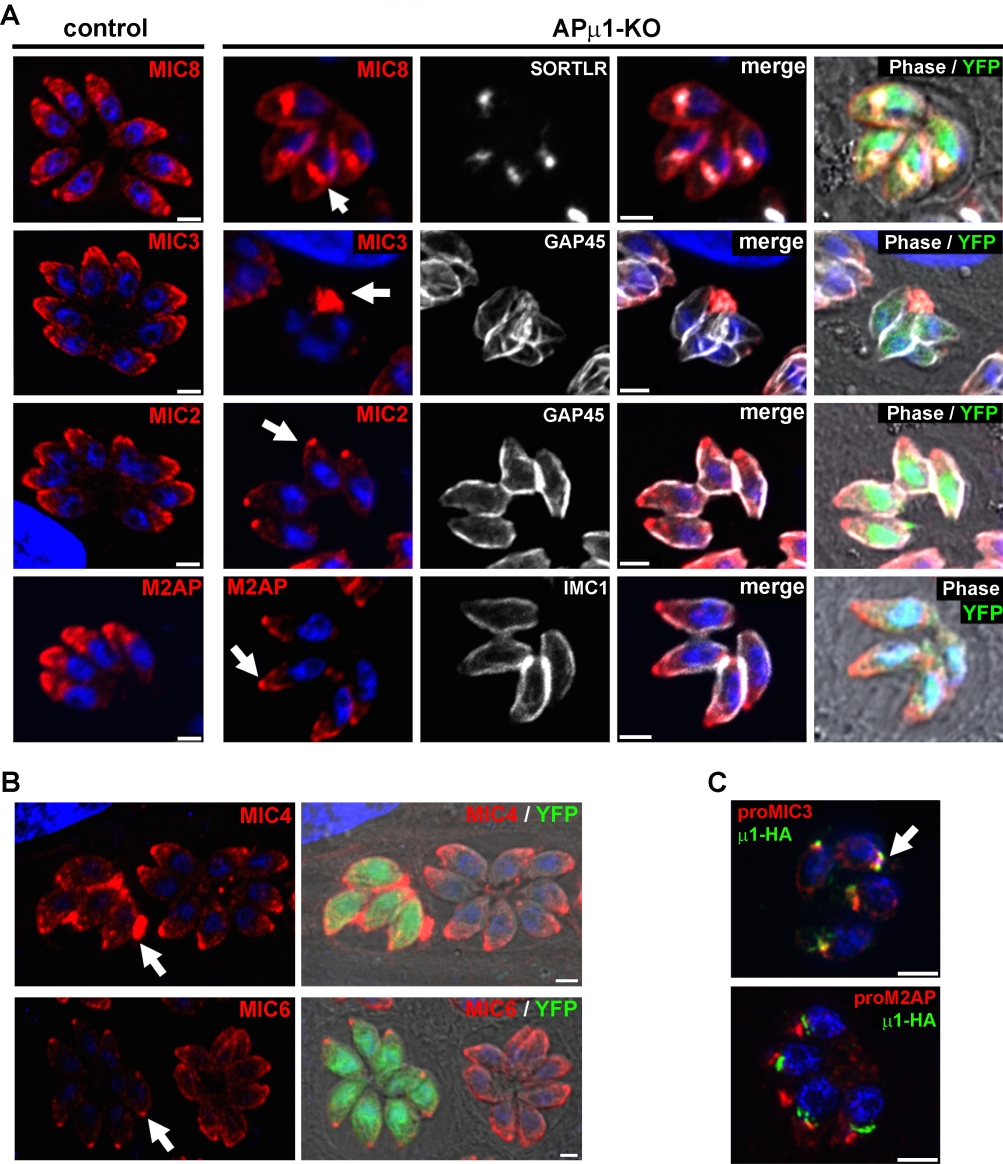
APμ1 ablation impairs microneme protein localization. **A**- Confocal images showing the localization of MIC8, MIC3, MIC2 and M2AP proteins (red) in control (YFP-negative parasites) and APμ1-KO parasites (YFP-positive). MIC8 accumulated in the parasite TGN, confirmed by its co-localization with SORTLR (white). MIC3 was found to be secreted into the parasitophorous vacuolar space (arrow). Parasite contours were stained with the IMC marker GAP45 (white). M2AP and MIC2 (parasite contours: IMC markers IMC1 and GAP45, respectively, both shown in white) displayed a preferential apical localization, while lateral micronemes were weakly detected. Nuclei are shown by DNA staining (blue). Bar: 2μm. **B-** Confocal microscopy images showing the localization of MIC4 (red, upper panel), and MIC6 proteins (red, lower panel) in control (YFP-negative parasites) and APμ1-KO parasites (YFP-positive). MIC4 was found to be secreted into the parasitophorous vacuolar space (arrow), while MIC6 was concentrated at the apex (arrow). **C**- SIM microscopy images showing the localization of μ1-HA (green) and proMIC3 (top) or proM2AP (bottom) in μ1-HA KI parasites. No co-localization between μ1-HA and proM2AP was observed in contrast to proMIC3. Bars: 2μm.

*Tg*AP1 had been previously shown to regulate rhoptry biogenesis at a post-Golgi level while microneme and dense granule formation was not perturbed [41]. Upon depletion of APμ1, we found that the soluble MIC3 protein was re-directed towards the vacuolar space, demonstrated by the GAP45 labeling of parasite contours, whereas MIC8 was found retained in the TGN, confirmed by its co-localization with SORTLR (Fig 4A). A weaker MIC8 staining was also detected at the plasma membrane suggesting that a part of the protein escaped by the constitutive secretory pathway to the parasite surface (Fig 4A). On the other hand, M2AP and MIC2 exit from the TGN was not impaired as the proteins were not seen retained in this compartment, nor secreted into the vacuolar space. However, both proteins appeared concentrated at the apex of the parasites, while lateral micronemes were weakly detected (Fig 4A). Similar to MIC2, the transmembrane MIC6 (Fig 4B) and AMA1 (S6A Fig) proteins, as well as MIC1 (S6A Fig), were found concentrated in apical micronemes, while the soluble protein MIC4 was found re-routed towards the vacuolar space (Fig 4B). Furthermore, in support of our data suggesting that *Tg*AP1 might not be involved in the MIC2/M2AP complex exit from the TGN, we observed by SIM microscopy that APμ1 co-localized with immature proMIC3 but not with immature proM2AP (Fig 4C). In addition, SORTLR was not mis-localized in APμ1-KO parasites (S6B Fig), showing that *Tg*AP1 likely functions downstream of SORTLR in the anterograde secretory pathway. Finally, we observed that dense granule biogenesis was not affected upon APμ1 ablation (S6C Fig). Therefore, our data indicate that *Tg*AP1 is part of the early sorting machinery that regulates distinct MIC protein complex transport from the TGN to their final destinations.

### *Tg*AP1 regulates rhoptry biogenesis

**Fig 5:**
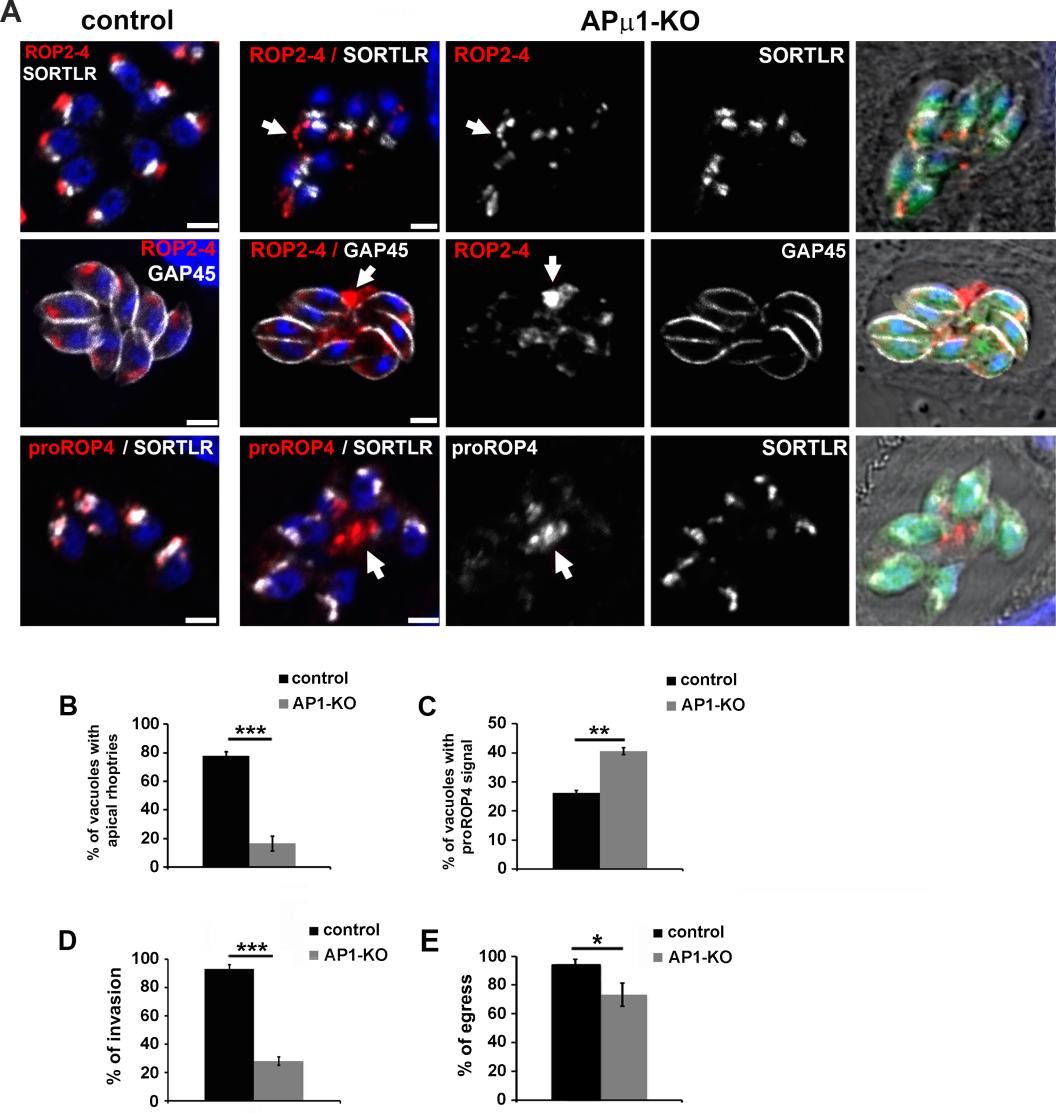
APμ1-KO parasites show defects in rhoptry formation. A-Confocal images showing the localization of ROP2-4 and proROP4 proteins (red) in control (YFP-negative vacuoles) and APμ1-KO parasites (YFP-positive vacuoles) together with the TGN marker SORTLR and the IMC marker GAP45 (both in white). In APμ1-KO parasites, mature rhoptries were found dispersed within the cytosol (upper panel, arrow) or in the vacuolar space (middle panel, arrow), while immature proROP4 proteins were found re-routed towards the vacuolar space and residual body (lower panel, arrow). Bar: 2μm. B- Histogram indicating the percentage of examined vacuoles displaying apically positioned rhoptries in control and APμ1-KO parasites. Mean values of three independent assays are shown ± SEM, ***p<0.001 (Student’s t-test). C- Histogram indicating the percentage of examined vacuoles positive for the immature protein proROP4 staining in control and APμ1-KO parasites. Mean values of three independent assays are shown ± SEM, **p<0.01 (Student’s t-test). D- Histogram depicting the percentage of invaded parasites after 45 min incubation with host cells of mechanically released parasites for control (YFP-negative) and APμ1-KO (YFP-positive) parasites. Mean values of three independent assays are shown ± SEM, ***p<0.001 (Student’s t-test). E- Histogram depicting the percentage of egressed vacuoles after induction with the calcium ionophore A23187 in control (YFP-negative) and APμ1-KO (YFP-positive) parasites. Mean values of three independent assays are shown ± SEM, *p<0.05 (Student’s t-test).

In YFP-positive parasites, we also observed that deletion of the APμ1 subunit drastically affected the formation of rhoptry organelles, which were detected as dispersed compartments distributed throughout the cell cytoplasm (Fig 5A, upper panel). We also found that ROP proteins were re-routed to the vacuolar space (Fig 5A, middle panel). Only 16.6 ± 4.7% of the examined vacuoles showed typical apically located club-shaped rhoptries in APμ1-KO parasites (Fig 5B). Next, we monitored immature pre-rhoptry compartment formation (Fig 5A and 5C). Quantification of parasites displaying a positive signal for proROP4 proteins indicated that 26.1 ± 1.2% of control parasites contained immature rhoptries, corresponding to dividing parasites in S/M phase of the cell cycle. In APμ1-KO parasites, an increase in the percentage of vacuoles positive for the proROP4 signal was counted (40.5 ± 1.2%), indicating no defect in ROP protein neosynthesis and suggesting a defect in ROP protein maturation. In agreement, immature proROP4 proteins were detected in the vacuolar space or at the residual body (Fig 5A, lower panel) in 71.8 ± 5.6% of the proROP4-positive vacuoles. Therefore, our data indicate that *Tg*AP1 is involved in immature proROP protein exit from the TGN compartment. In absence of APμ1, the neo-synthesized ROP proteins are secreted into the vacuolar space likely via the constitutive pathway, leading to an increase of vacuoles positive for proROP4 signal over the successive cell cycles in our unsynchronized parasite population. In addition, the presence of dispersed rhoptries containing mature ROP proteins (Fig 5A) may indicate an additional role of *Tg*AP1 in the rhoptry maturation process at a post-Golgi level. The fact that we observed both, the early proROP protein targeting to the vacuolar space and the later step of rhoptry organelle maturation defect, is likely a consequence of the unsynchronized nature of the APμ1 locus excision events together with the disappearance of the remaining endogenous protein, which might be effective at different stages of the rhoptry maturation process in this fast growing parasite.

Finally, we monitored host cell invasion and egress activity in APμ1-KO parasites. In agreement with the observed defect in rhoptry and microneme formation, we found that host cell invasion was drastically inhibited (Fig 5D). In contrast, egress was moderately impaired (Fig 5E), similar to what was observed for the Vps11-KO mutant [20].

### Over-expression of *Tg*APμ1 leads to defects only in rhoptry formation

**Fig 6:**
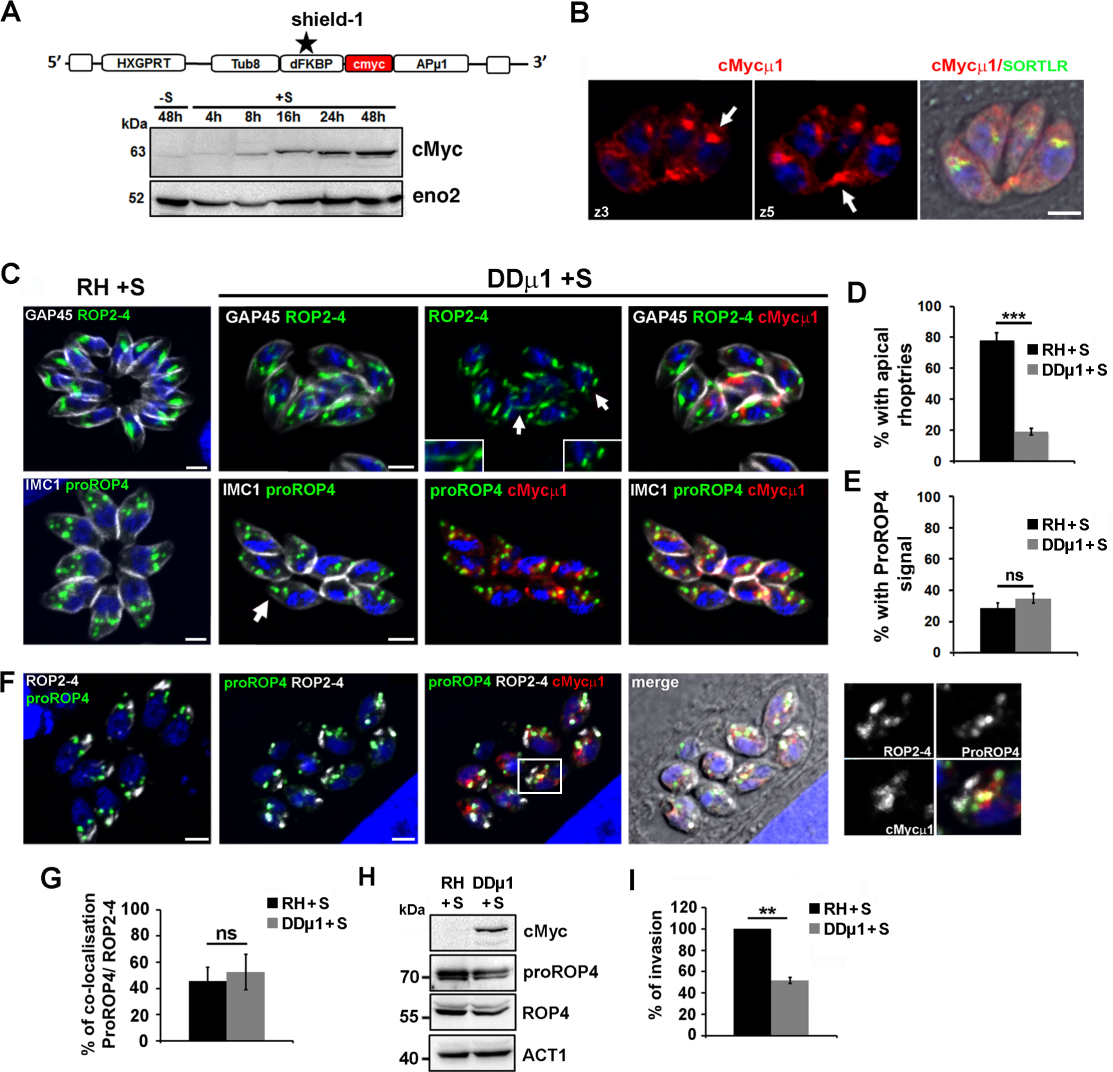
The inducible over-expression of APμ1 only perturbed rhoptry formation. **A-** Scheme showing the cloning strategy employed to insert the cMyc-tagged μ1 subunit under the influence of the destabilisation domain ddFKBP (DD). After addition of the synthetic ligand shield-1, the protein is no longer degraded but accumulated in the parasites (Tub8: tubulin promotor; HXGPRT: resistance cassette). The WB image shows the accumulation of the cMycμ1 protein upon shield-1 treatment for the indicated time periods. The protein eno2 was used as a loading control. **B-** Confocal microscopy images showing the localization of the over-expressed cMycμ1 protein (red) in a clonal population of DDμ1 parasites in two confocal planes (z3 and z5). cMycμ1 was detected at the Golgi apparatus (co-localization with SORTLR shown in green in the merged image) and in vesicles accumulating at the basal pole of the parasites (arrows). Bar: 2μm. **C-** Confocal images showing the localization of ROP2-4 (upper panel) or proROP4 (lower panel) proteins (both in green) and cMycμ1 (red) in control RH and DDμ1 parasites incubated with shield-1 (+S) for 24 hours. The contours of the parasites are delineated by staining of the IMC markers, GAP45 or IMC1 (white). Rhoptries are detected as dispersed atrophied compartments (arrows and insets in upper panel) in DDμ1 parasites, while proROP4 compartments were normally formed (arrow in lower panel). **D**- Histogram indicating the percentage of examined vacuoles displaying apically positioned rhoptries in control and DDμ1 parasites induced with shield-1 (+S) for 24 hours. Mean values of three independent assays are shown ± SEM, ***p<0.001 (Student’s t-test). **E**- Histogram indicating the percentage of vacuoles positive for the immature protein proROP4 staining in control and DDμ1 parasites induced with shield-1 (+S) for 24 hours. Mean values of three independent assays are shown ± SEM. **F-** Confocal images showing the co-localization of ROP2-4 (white), proROP4 (green) and cMycμ1 (red) in control RH and DDμ1 parasites incubated with shield-1 for 24 hours. Bar: 2μm. On the right, a zoom of the Golgi region indicated by a white frame in the merge image is shown. **G-** The histogram indicates the percentage of co-localization between the proROP4 signal and the ROP2-4 signal after image acquisition by airyscan confocal microscopy. Data are indicated as average ± SD, n = 15 vacuoles. **H-** WB analysis of ROP4 protein proteolytic processing in control RH and DDμ1 parasites incubated with shield-1 (+S) for 24 hours. No defect was found as the immature proROP4 and mature ROP4 proteins were detected at similar amounts in both parasites lines. Actin (ACT1) was used as a loading control and the detection of the cMycμ1 protein was used as a control for the shield-1 induction. **I-** Invasion assay. Histogram depicting the percentage of invaded parasites after 45 min incubation with host cells of mechanically egressed parasites for both the parental strain and DDμ1 parasites induced with shield-1 (+S) for 16 hours. Mean values of three independent assays are shown ± SEM, **p<0.01 (Student’s t-test).

It has been previously shown that over-expression of APμ1 or a point-mutated form of APμ1 caused a drastic defect in rhoptry formation at the post-Golgi level, while microneme biogenesis was not impaired [41]. To understand the distinct observed phenotypes compared to the APμ1-KO parasites, we applied a similar strategy and conditionally over-expressed the μ1 subunit (DDμ1 parasites) using the ddFKBP system [49]. Western blot analysis confirmed the time-dependent accumulation of the cMyc-tagged μ1 subunit upon shield-1 addition in the growth medium (Fig 6A). By IFA, the over-expressed cMycμ1 protein was detected at the Golgi area, but also in cytoplasmic vesicles particularly concentrated at the basal pole of the parasite (Fig 6B). We found that cMycμ1-overexpressing parasites displayed less pronounced defects compared to APμ1-KO parasites. First, MIC2/M2AP and MIC8/MIC3 complexes were both correctly targeted to micronemes (S7A Fig) as previously observed after over-expression of the mutated μ1D176A subunit [41], and MIC protein processing was not affected (S7B Fig). In contrast to micronemes, rhoptry formation was perturbed, and ROP proteins were mostly detected in dispersed compartments throughout the parasite cytoplasm (Fig 6C, insets). Only 19.0 ± 2.1% of the population showed apically localized, mature rhoptries (Fig 6D). Furthermore, in agreement with the observed defect in mature rhoptry formation, a partial decrease in host cell invasion was monitored (Fig 6I). However, in opposite to APμ1-KO parasites, in DDμ1-induced parasites, proROP4 proteins were not re-routed to the vacuolar space or into the residual body, and the percentage of proROP4 positive vacuoles was similar to control parasites (Fig 6C and 6E). Furthermore, our analysis by confocal microscopy indicated that proROP4 proteins exit normally from the SORTLR-positive TGN compartment (S7C and S7D Fig), as the percentage of co-localization between these two compartments did not vary compared to control parasites. In addition, we also monitored no change in the percentage of co-localization between immature proROP4 and mature ROP2-4 proteins (Fig 6F and 6G) suggesting no defect in ROP protein processing, which was also confirmed by WB analysis (Fig 6H). Of note, we could also observe a co-localization between proROP4-positive compartments and the over-expressed cMycμ1 protein (Fig 6F, insets). To verify that the over-expression of the μ1 subunit induced defects in rhoptry formation via a *Tg*AP1-dependent activity and not indirectly by titrating other μ1-interacting factors, we performed an IP of the cMycμ1 protein. Immunoprecipitation of cMycμ1 using anti-cMyc antibodies identified the three other subunits of the *Tg*AP1 complex and *Tg*EpsL as the main protein partners (Table 3 and S8 Fig). In addition, APσ1-HA correctly localized at the TGN (S7E Fig) suggesting that cMycμ1 over-expression does not cause the mis-location of the endogenous *Tg*AP1 complex. These data suggest that the over-expressed cMycμ1 protein likely integrates into a functional AP1 complex. In summary, we found that over-expressed APμ1 impaired the post-Golgi maturation process of rhoptry organelles without perturbing proROP protein proteolytic processing, thus acting at a similar or posterior step to this event in the secretory pathway. We therefore examined the morphology of the endosome-like compartment in DDμ1 parasites.

**Table 3:** List of proteins identified by mass spectrometry following the IP of cMycμ1 protein in cMycμ1-overexpressing parasites induced with shield-1 for 24 hours. The detailed list is included in S8 Fig. The parental strain RH was used as a control for non-specific binding to the antibody-coated beads (no peptides corresponding to the indicated proteins were found in the control IP). The table indicates the number of unique peptides and spectra for each identified protein.

Protein name	accession numbers	molecular weight (Da)	Number of peptides	Number of spectra
Gamma 1 adaptin	TGGT1_313670	107 036	49	189
beta adaptin	TGGT1_240870	101 920	51	160
Sigma1 adaptin	TGGT1_270370	19 679	9	63
ENTH domain-containing protein	TGGT1_214180	65 903	4	4

### Over-expression of *Tg*APμ1 perturbed the Rab5A-positive ELC morphology

**Fig 7:**
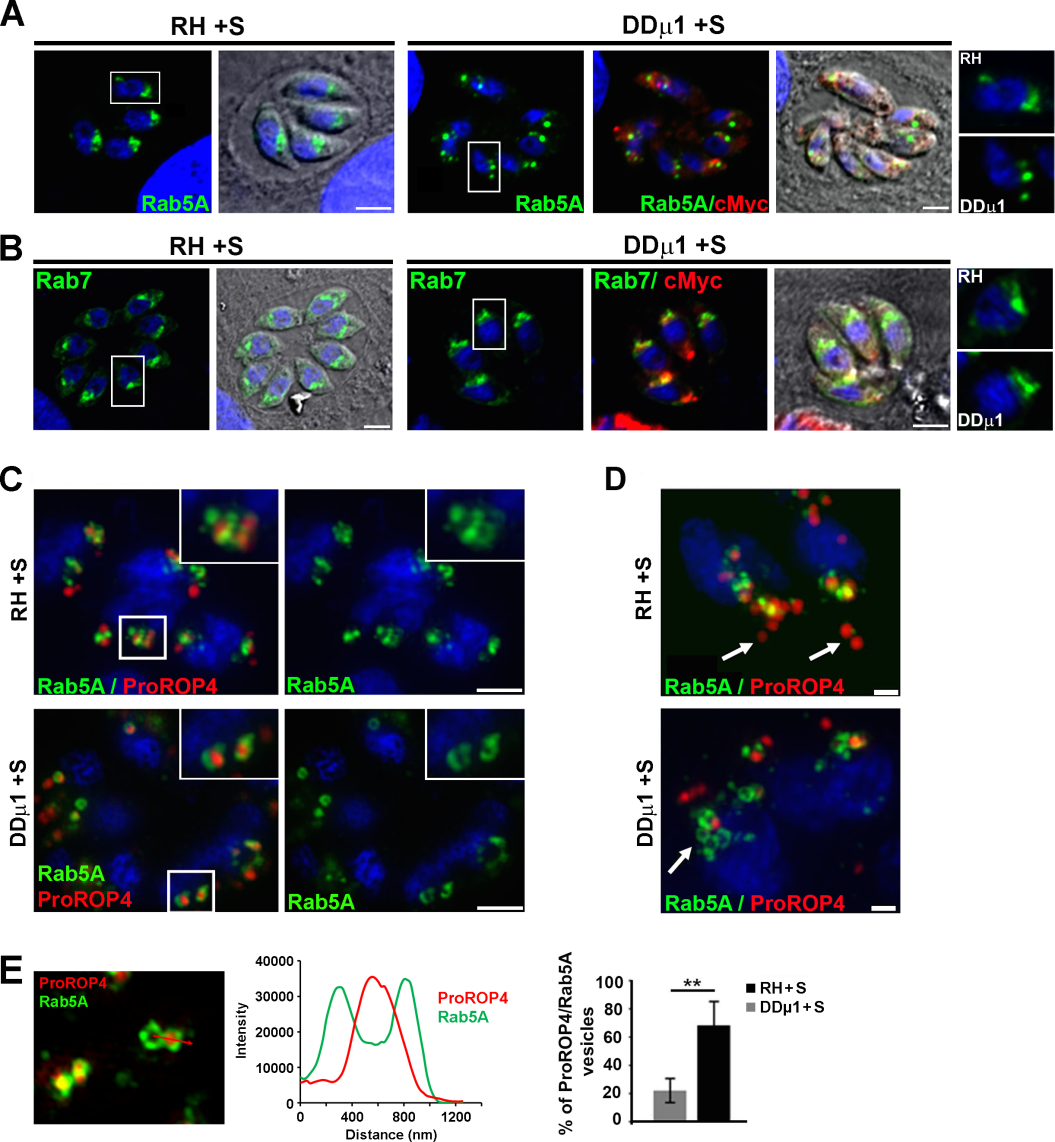
Over-expression of APμ1 perturbed the Rab5A compartment morphology. **A, B**- Confocal images showing the localization of Rab5A (A, green), Rab7 (B, green) and cMycμ1 (A, B, red) in the parental strain RH and DDμ1 parasites treated with shield-1 (+S) for 16 hours. A zoom of the Golgi area indicated by the white frame in each image is shown as an inset on the right. Bar: 2μm. **C-** SIM images showing the localization of proROP4 (red) and Rab5A-HA (green) proteins in control RH parental strain (upper panel) and cMycμ1 over-expressing parasites (lower panel) treated with shield-1 (+S) for 16 hours. Bars: 2μm. **D-** SIM images showing the co-distribution of Rab5A-positive vesicles surrounding proROP4 vesicular compartments in RH parental strain (upper panel), while TGN-distant preROP compartments were negative for Rab5A staining (arrows). Induced DDμ1 parasites exhibited Rab5A-positive enlarged vesicular compartments (lower panel, arrow) empty of proROP4 proteins (red). Bars: 500 nm. **E-** Left: SIM image of DDμ1 parasites induced with shield-1 showing proRO4 proteins (red) contained in vesicles with a strong Rab5A (green) signal at their limiting membrane illustrated by the intensity profile of each signal (graph). Right: Histogram depicting the percentage of proROP4-positive vacuoles showing a Rab5A signal at their limiting membrane in RH and DDμ1 parasites treated with shield-1 (+S). Data are presented as average ± SD (n = 50 parasites), ***p<0.001 (Student’s t-test).

We transiently transfected HA-tagged Rab5A and Rab7 in DDμ1 parasites and examined the distribution of the ELC compartment after shield-1 induction (Fig 7A and 7B). Interestingly, we found that the Rab5A compartment displayed an altered morphology, appearing as large vesicular structures scattered along the Golgi area in comparison to the more homogenous and continuous distribution observed in control parasites (Fig 7A). In contrast, the Rab7 compartment did not show detectable changes by confocal microscopy after overexpression of cMycμ1 (Fig 7B). Therefore, we next examined in details the relationship between the Rab5A-positive compartment and the ROP maturation process using SIM microscopy. In control parasites, at the onset of ROP protein neo-synthesis during parasite division, preROP compartments seemed to emerge from the TGN as already formed large vesicular compartments co-distributing with Rab5A-positive vesicles or displaying a faint Rab5A signal at their limiting membrane (Fig 7C, upper panel). More distant preROP compartments from the TGN, likely en route to the apical pole of the parasite, were detected as Rab5A-negative compartments (Fig 7D, upper panel, arrows) suggesting that the maturation process involves a transient step of transport through the Rab5A-positive ELC. In DDμ1 induced parasites, proROP4 proteins were found retained in large vesicular compartments emerging from the Golgi/ELC, which displayed a strong Rab5A signal at their limiting membrane (Fig 7C, lower panel). We calculated that 63.6 ± 18.2% of proROP4-positive preROP compartments were also positive for Rab5A compared to only 21.9 ± 13.0% in control parasites (Fig 7E). Notably, the formation of large and dispersed Rab5A-positive compartments empty of proROP4 proteins could also be observed in DDμ1 induced parasites (Fig 7D, lower panel) and not in control cells. This observation suggests a more general process of AP1-dependent regulation of Rab5A-positive endosomal membrane dynamics apart from its contribution in the process of preROP compartment maturation.

Collectively, our data suggest that the maturing preROP compartments require a *Tg*AP1-dependent activity to further evolve towards the mature apically anchored club-shaped organelles. Importantly, this *Tg*AP1 activity seems to be tightly connected to the activity of the Rab5A-positive ELC. Therefore, the study of the less severe phenotype associated with over-expression of the APμ1 subunit allowed us to identify an additional step of *Tg*AP1-mediated regulation of the rhoptry maturation process at the level of the endosome-like compartment.

Altogether, our data indicate that *Tg*AP1 acts at different trafficking steps during secretory organelle biogenesis. First, *Tg*AP1 regulates the anterograde transport of the studied microneme complexes from the TGN. Second, our data suggest that *Tg*AP1 regulates rhoptry formation by acting on both, at the level of immature ROP protein exit from the TGN as well as at the ELC level to ensure the rhoptry maturation process into apically anchored, club-shaped organelles.

### *Tg*AP1 regulates cell division

**Fig 8:**
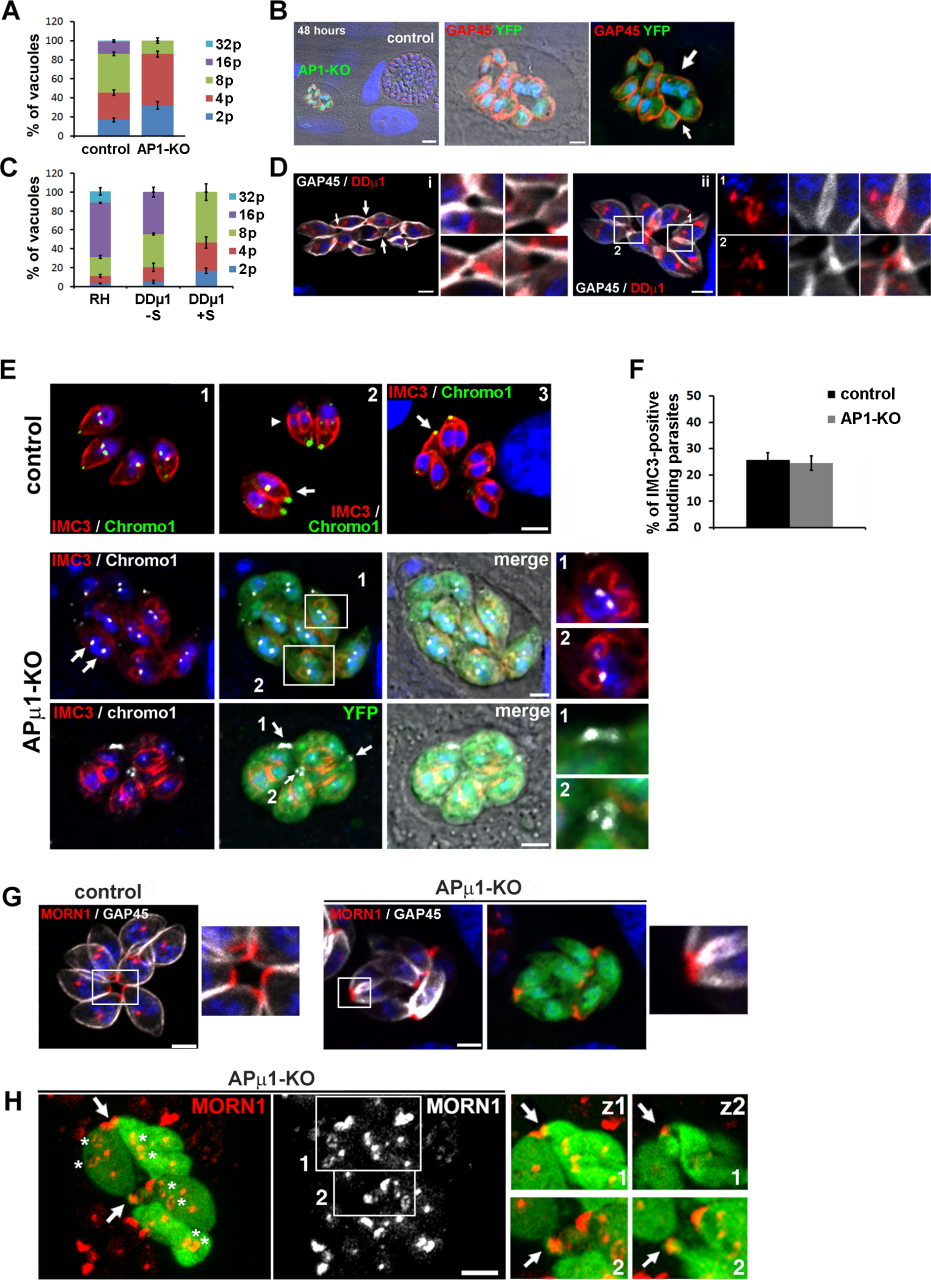
*Tg*AP1 regulates parasite growth. **A, C:** Intracellular growth assay performed in APμ1-KO parasites after rapamycin treatment (A) and DDμ1 parasites after ± shield-1 induction (C) at 24 hours post-invasion revealed defects in parasite replication. The histograms depict the percentage of vacuoles containing 2, 4, 8, 16 or 32 parasites. Mean values of three independent assays are shown ± SEM. **B, D**: Confocal images showing the disorganized appearance of dividing APμ1-KO (B) and DDμ1 parasites (D). B- After 48 hours of growth, the APμ1-KO parasites (YFP-positive, green) have stopped to grow and parasites seem to display a defect in segregation. Note the apparent rupture of the cortex revealed by the GAP45 staining (red) (arrow). Nuclei are shown by DNA staining (blue). **D-** Induced DDμ1 parasites were labeled for GAP45 (white) and overexpressed cMycμ1 proteins (red). Left (i): Mis-organised vacuole showing tethered parasites with an apparent defect in lateral segregation revealed by the GAP45 staining (insets: zoom of the different areas indicated by arrows in the main image). Right (ii): Confocal images showing the enrichment of the overexpressed cMycμ1 protein at sites, where parasites have remained tethered to each other (insets: region 1: lateral sides, region 2: basal pole). **E**- Upper panel: Confocal microscopy images in control parasites showing the duplication of the centromers (centromeric protein chromo1, green, image 1) and the formation of daughter buds (IMC3 protein, red, images 2, arrowhead). Note that chromo1 transiently accumulates at the basal pole of parasites at the very end of the daughter cell budding process (image 3, arrow). Lower two panels: Confocal microscopy images of APμ1-KO parasites (YFP-positive parasites) showing the duplication of the centromers (chromo1, white arrows) and the formation of daughter buds (IMC3, red, middle and lower panels). Note the accumulation of chromo1 at sites connecting mother parasites, while daughter cells complete bud formation (lower panel, arrows). Bars: 2μm. **F**- Histogram depicting the percentage of vacuoles containing budding daughter cells (IMC3 staining) in control and APμ1-KO parasites. Mean values of three independent assays are shown ± SEM. **G-** Airyscan confocal microscopy images showing the localization of MORN1-cherry (red) in control (YFP-negative, left) and APμ1-KO parasites (YFP-positive, right) parasites. The parasite contours were delineated by staining the IMC marker GAP45 (white). The insets show a zoom of the region indicated by a frame in the main image. **H**- Airyscan confocal images showing APμ1-KO parasites (YFP-positive, green) expressing the MORN1-cherry protein (red). MORN1-positive daughter rings were normally assembled (asterisks) but mother parasites seemed to be attached by their basal pole (arrows), which appeared as deformed elongated membranous structures (insets: two confocal planes z1 and z2 of the regions 1 and 2 indicated with a white frame in the main image, arrows). Bars: 2μm.

When observed by IFA, both the induced DDμ1 and APμ1-KO parasites display a clear growth defect. Quantification indicated a delay in parasite growth starting from stage 4 to 8 and 8 to 16 parasites per vacuole in APμ1-KO and DDμ1 parasites, respectively (Fig 8A and 8C). In particular, after 48 hours, the APμ1-KO parasites appeared to have stopped dividing compared to neighboring YFP-negative vacuoles (Fig 8B). The growth defective parasites seemed to be tethered by lateral contact sites or even fused together. Parasites that have broken out were also often observed (Fig 8B, arrows). Very similar but milder morphological defects were detected in DDμ1 parasites (Fig 8D). After the stage with 8 parasites per vacuole, the parasites were no longer organized in a rosette–like structure and also appeared “tethered” by their lateral sides or basal pole (Fig 8D, i, arrows and insets). Importantly, the over-expressed cMycμ1 protein was found enriched at sites of lateral contacts between parasites (Fig 8D, ii, region 1, insets) and at their basal pole (Fig 8D, ii, region 2, insets).

**Fig 9:**
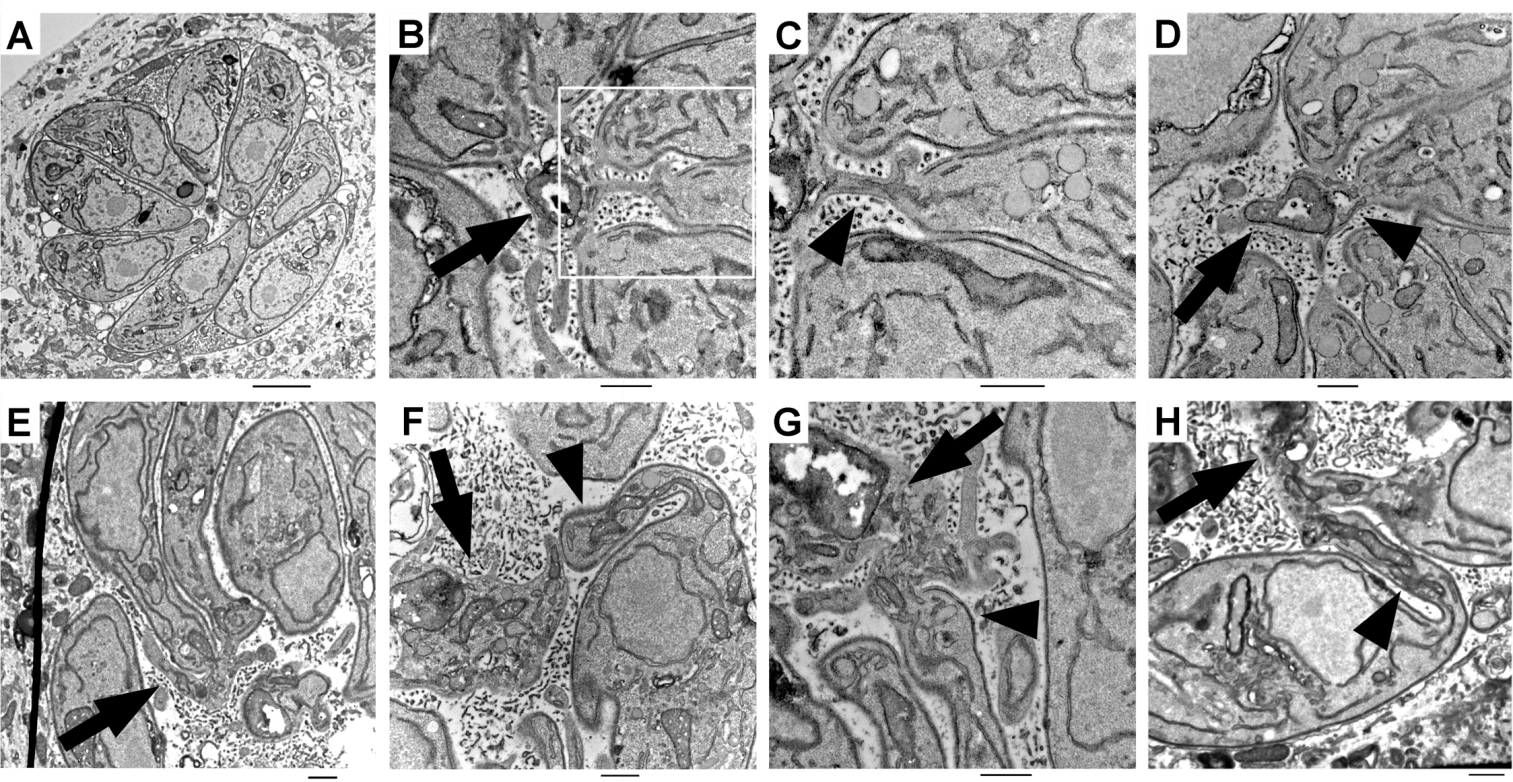
Correlative Light Electron Microscopy (CLEM) images illustrating the organization of the basal pole (arrow heads) and the residual body (arrows) in control and APμ1-KO parasites. **A-D**: Non-YFP control vacuoles (**C**: zoom of the region indicated by a white frame in **B)**. Note the typical organization in a rosette-like structure and the correct morphology of the parasites. **E-H**: YFP-positive APμ1-KO parasites (detected in the region 3T shown in S10 Fig). Bars: 500 nm.

These observations suggest that the *Tg*AP1-defective parasites suffer from a defect of division at the late stages of cytokinesis when daughter cells segregate. To verify this hypothesis, we examined in detail the different steps of the division process in these parasites. In APμ1-KO parasites, we found that centromers replicate normally (Fig 8E, middle panel). New daughter buds positive for the inner membrane complex (IMC) marker IMC3 were also assembled (Fig 8E, middle and lower panel) and the APμ1-KO parasites displayed a similar percentage of IMC3-positive budding daughter cells compared to control parasites (Fig 8F). Similar observations were obtained in DDμ1 parasites (S9 Fig). Interestingly, while labeling for the centromeric protein chromo1, we noticed that APμ1-KO parasites, which had almost completed their budding process, displayed a chromo1 localization at the basal pole in a region that seemed to connect mother parasites (Fig 8E, lower panel, arrows and insets). The dynamic nature of the chromo1 protein localization during the different stages of the cell cycle has been previously described [50]. We confirmed this localization by IFA in control parasites (Fig 8E, upper panel) and found that the protein also transiently localized at the basal pole of the mother parasite while daughter parasites terminated the budding process (Fig 8E, upper panel, image 3, arrow). Interestingly, this chromo1 dynamic localization is somehow reminiscent of the Chromosomal Passenger Complex (CPC) in higher eukaryotes, which translocates from the spindle pole in M phase to the mid-body at the end of cytokinesis [51]. This observation again suggested that the division defect in APμ1-KO parasites likely occurs at the late stage of parasite segregation. To investigate further this hypothesis, we examined the localization of the basal complex protein MORN1, which forms a contractile ring required to segregate nascent daughter cells [52]. We transiently expressed MORN1-cherry in APμ1-KO parasites and monitored its localization both in fixed cells and by live imaging (Fig 8G and 8H and S3 Movie). We were able to visualize the formation of MORN1-cherry positive contractile rings at the basal pole of nascent daughter parasites, suggesting that basal complex assembly is not impaired in APμ1-depleted parasites (Fig 8H and S3 Movie). However, in opposite to control parasites where MORN1-positive basal complexes were clearly seen separated one from another, in APμ1-KO parasites, the mother parasites appeared to remain connected by their MORN1-positive basal pole (Fig 8G and 8H, insets: region 2). When examined more carefully, we observed that the basal complex constriction seemed prolonged by unusual elongated and wider membranous structures (Fig 8H, insets: region 1). Interestingly, we were also able to visualize similar deformed elongations of the parasite basal pole after performing Correlative Light Electron microscopy (CLEM) in APμ1-KO YFP-positive parasites (Fig 9 and S10A Fig). In control parasites, the residual body appeared as a well-organized structure, in which each parasite basal pole displayed a discrete constricted region connecting the parasites to a central mass of membrane, previously described as the remnant of the mother cell formed at the end of cytokinesis (Fig 9A–9D). In APμ1-KO parasites, the basal pole of each parasite appears elongated, wider and connected to the residual body in a disorganised manner (Fig 9E–9H). In addition, while control parasites displayed a typical rosette-like organization, APμ1-KO parasites exibited a general deformed morphology that could indicate a defect in their cortical integrity (Fig 9E). In more drastically affected vacuoles, parasites appeared to have broken out or to remain connected by their lateral sides (S10B Fig, images e and f). In the latter case, the pellicles from neighbouring daughter cells appeared to not have been fully segregated one from another, ressembling the parasite division defects observed in Rab11A defective parasites [53]. Supporting the hypothesis that APμ1-KO parasites suffer from a segregation blockage, we noticed that when egress was artificially triggered in APμ1-KO parasites by incubation with the calcium ionophore A23187, most of the monitored vacuoles initiated the process (Fig 5E) demonstrated by the visualization of some parasites escaping the vacuole, however, the other parasites remained in the vacuole and appeared attached together, unable to escape (S4 Movie). Finally, in agreement with our IFA observations (Fig 5), APμ1-KO parasites did not possess apically localized mature rhoptries and only apical micronemes were detected (S10B Fig, images c and d).

**Fig 10:**
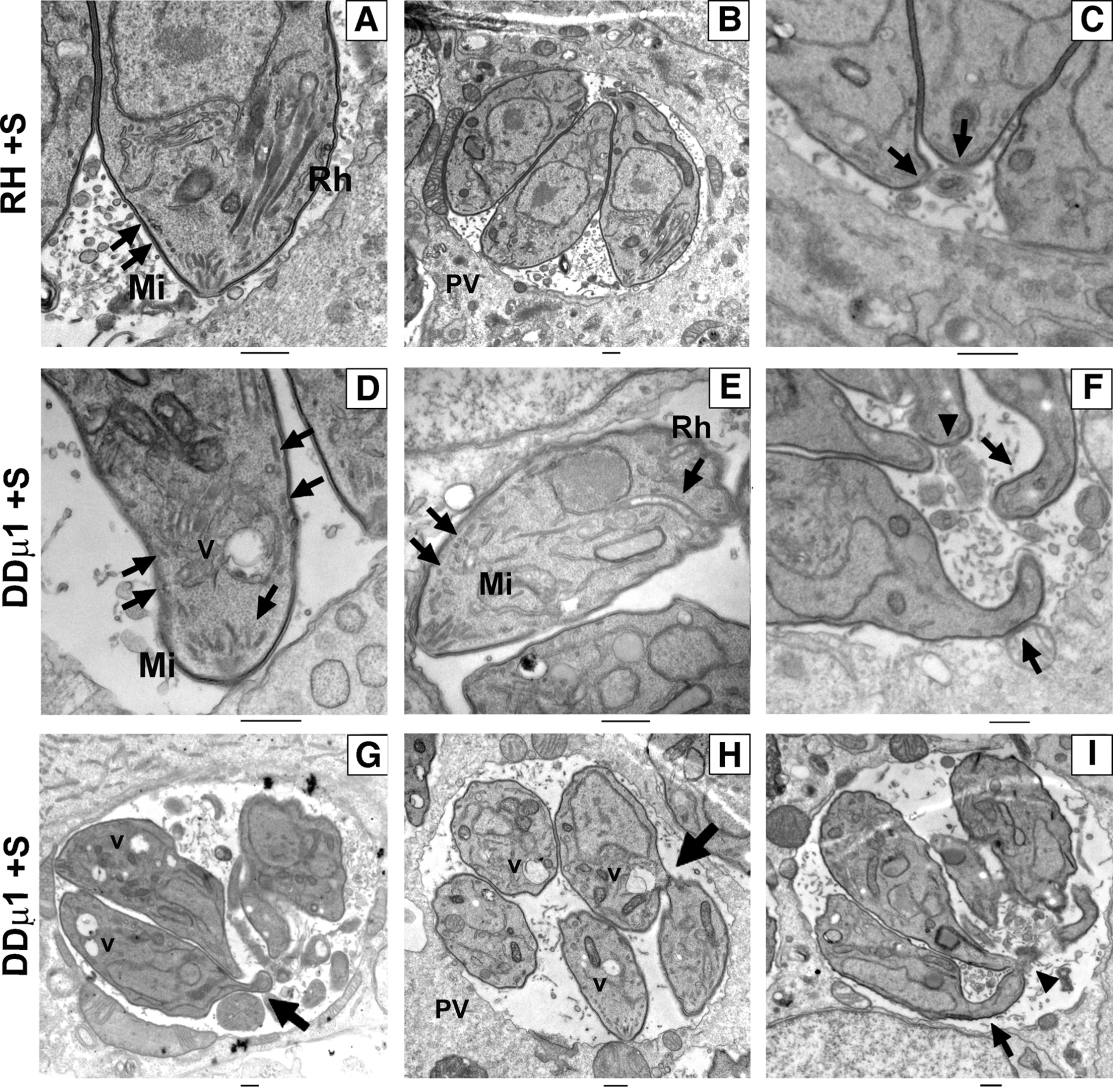
Transmission electron microscopy images showing the formation of mature rhoptries (Rh) and micronemes (Mi) anchored at the apical pole in control parasites (**A**) and the normal distribution of parasites in rosette-like structures (**B**). Bars: 500nm. (**C**)- Zoom of the posterior end of the control parasites showing the tight constriction of the basal pole with a thin continuity to the residual body (arrowhead). In DDμ1 parasites induced with shield-1 (+S) for 24 hours (D-I), apically positioned rhoptries could not be detected in contrast to micronemes (**D**) or they were found dispersed in the cytoplasm (**E,** arrow). Numerous giant lucent vesicles (V) were also observed (D, G, H). In addition, the parasites were found disorganised within the vacuole with a distorted morphology (G-I) particularly at the basal pole (F, G and I, arrow), which appeared deformed and elongated (arrows in F) despite the detection of the residual body (F and I, arrowhead). In addition, some parasites seemed to remain attached by discrete lateral contact sites (H, arrow). Bars: 500nm. Mi: micronemes, Rh: rhoptries, PV: parasitophorous vacuole, V: vesicles.

We also performed transmission electron microscopy (TEM) in induced DDμ1 parasites. We found that the parasite morphology was similarly affected, together with parasite organization within the vacuole (Fig 10G–10I), in opposite to control parasites (Fig 10A and 10B), which were organized in a rosette-like structure. Similar to APμ1-KO parasites, the basal pole of the DDμ1 parasites displayed a pronounced change in their morphology (Fig 10F, 10G and 10I, arrows). In contrast to control cells (Fig 10C), our images suggest that DDμ1 induced parasites were unable to properly constrict their basal extremity, which appeared deformed with wide elongations (Fig 10F and 10G). In addition, we also observed parasites that were attached by discrete lateral contact sites (Fig 10I, arrow). Furthermore, in agreement to our IFA images (Fig 4 and Fig 5), in DDμ1 induced parasites, apically anchored mature rhoptries were not detected or present as dispersed compartments throughout the cell (Fig 10D and 10E), while apical and lateral micronemes were still visualized (Fig 10D). The parasites also accumulated large lucent vesicles of unknown nature in their cytoplasm (Fig 10D, 10G and 10H). We could detect membranous or vesicle-like structures within their lumen and some of our images suggest that they could be formed by internal budding of the limiting membrane (S11 Fig), as previously observed for the VAC compartment [54]. However, presently, we cannot conclude whether these vesicles represent dispersed and fragmented VAC-related compartments or enlarged Rab5-positive endosomal structures that we detected by SIM microscopy (Fig 7).

Collectively, our data suggest that *Tg*AP1 is involved in parasite division by regulating the very late stages of cytokinesis after the budding process has been completed. Though we did not directly demonstrate it, our data converge towards the hypothesis that a *Tg*AP1-dependent delivery of vesicles at the plasma membrane, directly from the Golgi, or indirectly via a recycling activity of the mother cell plasma membrane from the residual body, is required to complete daughter cell segregation. By this activity, apart from its role in ROP and MIC protein transport, *Tg*AP1 could deliver lipids as well as important regulatory factors, such as regulators of cytoskeleton dynamics. These hypotheses are supported by our observations that the over-expressed cMycμ1 protein was found accumulated at the basal pole of dividing parasites and lateral contact sites (Figs 6B and 8D) and that endogenous *Tg*AP1 was also detected in numerous peripheral vesicles at the parasite cortex in APμ1-HA KI parasites (Fig 1D). Of note, such as illustrated in Fig 1B, we also noticed in some vacuoles a very discrete localization of endogenous APμ1-HA at a point connecting the basal pole of the parasites.

## Discussion

### Role of *Tg*AP1 in ROP and MIC protein trafficking

**Fig 11:**
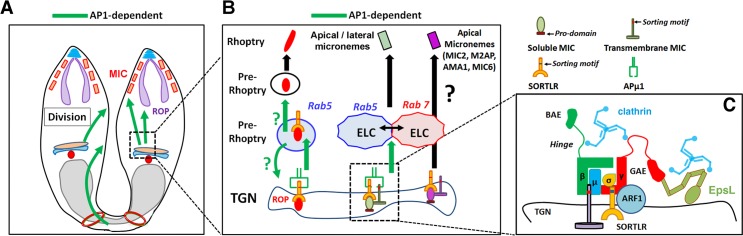
Model summarizing the different functions of AP1 in *T*. *gondii*. **A**- Our data indicate that *Tg*AP1 is involved in the sorting of MIC proteins from the TGN and rhoptry biogenesis, as well as, participates to daughter cell segregation. This latter activity might be regulated by a *Tg*AP1-dependent recycling activity of the mother plasma membrane from the residual body or a direct transport of vesicles from the Golgi to the nascent daughter pellicles. **B**- *Tg*AP1 regulates the sorting and transport of all the different studied MIC protein complexes from the TGN (green arrow), including MIC3/8, MIC1/4/6 and M2AP/MIC2, resulting in the loss of lateral micronemes containing these proteins. However, a subpopulation of apical micronemes, containing the proteins MIC2/M2AP, AMA1 and MIC6, were still detected upon APμ1 ablation. At the molecular level, one can envision that *Tg*AP1 recognizes via its subunits γ‒σ, the dileucine motif present in the cytoplasmic tail of SORTLR, which has loaded the soluble MIC partner (such as MIC3). *Tg*AP1 could simultaneously bind to the tyrosine motif of the transmembrane MIC partner (such as MIC8) via its subunits β‐μ, thereby participating to the complex stabilization and transport into clathrin-coated vesicles from the TGN. These putative sorting mechanisms have to be confirmed. Finally, we found that *Tg*AP1 regulates ROP protein transport from the TGN to the Rab5A-positive ELC and also the subsequent steps of the rhoptry maturation process. *Tg*AP1 could regulate the latter activity either, by stimulating ROP protein exit from the ELC or by retrieving material from the preROP compartments in a Rab5A-dependent manner to ensure the following steps of maturation into club-shaped apically anchored organelles (green arrows). The Rab7-positive ELC also likely participates in ROP and MIC trafficking, though a specific functional relationship was found between *Tg*AP1 and the Rab5A-positive ELC. **C**- Our data also indicate that the AP1 complex in *T*. *gondii* functions as a conserved heterotetrameric complex composed of the μ1, σ1, γ and β subunits and interacts with ARF1 and clathrin. We also found that the ear appendage domain of the γ subunit associates with the unique ENTH-domain containing protein *Tg*EpsL.

Previous studies have shown that MIC proteins navigate through the secretory pathway as complexes presumably assembled at the TGN level, which include one transmembrane escorter and one or two soluble partners. For example, MIC6 escorts MIC1/MIC4 and MIC8 associates with MIC3. All the members of the complex possess sorting signals required to address the proteins to the mature organelles, in particular the prodomain of the soluble partner and the cytoplasmic domain of the transmembrane partner. After depletion of the MIC6-CD containing sorting signals, both MIC1 and MIC4 were retained together with MIC6ΔCD in the ER/Golgi [17]. The prodomain of MIC3 was shown to be essential for complex transport through the secretory pathway [14], however, deletion of MIC8 did not impact the targeting of MIC3 to micronemes, suggesting distinct regulatory trafficking mechanisms [16]. In APμ1-KO parasites, soluble MIC3 was re-directed towards the vacuolar space, whereas the transmembrane MIC8 protein was mainly retained in the TGN. Interestingly, we also found that similar to MIC3, the soluble MIC4 protein was re-routed to the vacuolar space; however the transmembrane MIC6 protein was not retained in the Golgi but localized to a sub-population of apical micronemes. Therefore, this data suggests that these two MIC complexes exhibit different trafficking mechanisms in relation to *Tg*AP1 function, MIC8 being the only transmembrane MIC protein that we found retained in the Golgi upon APμ1 ablation. We also observed that MIC2 and AMA1 were localized in apical micronemes while the typical lateral staining was weakly detected. However, in opposite to MIC4 and MIC3, the soluble M2AP was not re-routed to the vacuolar space but accumulated together with MIC2 in apical micronemes. This result shows that despite the lack of lateral MIC2/M2AP containing micronemes, both proteins of the complex likely exit the Golgi and are targeted to apical micronemes. Presently, we cannot conclude whether this loss is due to an indirect effect on parasite cortical integrity, which could impair lateral anchoring of these organelles but not anchoring to the conoid region, or caused by a direct effect on protein trafficking. In addition, the MIC1/MIC4/MIC6 complex was dissociated in absence of *Tg*AP1 similarly to the MIC3/MIC8 complex, supporting the hypothesis that *Tg*AP1 might play an important role in complex stabilization at the TGN before their export. Of note, SORTLR has been shown to interact with the soluble proteins MIC1, MIC4 and MIC5 and to be involved in the trafficking of the corresponding MIC1/4/6 complex and the MIC5 protein, while interaction with transmembrane MIC proteins has not been investigated. However, the MIC3/MIC8 and MIC2/M2AP complexes were also mis-targeted. Therefore, further studies are now required to understand better at the molecular level, whether *Tg*AP1 interacts with both components of the sorted MIC complex, which could include SORTLR loaded with the soluble MIC as well as the associated transmembrane MIC protein (see proposed model in Fig 11) by their respective sorting signals. To address this question, it will be interesting to investigate the role of the dileucine motif present in the cytoplasmic tail of SORTLR in the differential sorting of soluble versus transmembrane MIC proteins at the TGN.

Concerning the role of *Tg*AP1 in ROP protein trafficking, our data indicate that immature proROP proteins were re-directed towards the vacuolar space and the basal body in APμ1-KO parasites, indicating an *Tg*AP1-dependent transport in the anterograde secretory pathway before their proteolytic processing occurs, therefore most likely at the TGN level. In contrast, perturbing *Tg*AP1 functions by an inducible over-expression of the μ1 subunit led to less severe effects. MIC proteins were not mis-sorted but maturing rhoptries were retained in Rab5A-positive compartments and the formation of apically anchored club-shaped rhoptries was impaired. Of note, a defect in both, the anterograde and retrograde transport of proteins from the Rab5A-positive endosomes would lead to an accumulation of membrane and disorganization of these compartments. Presently, we cannot exclude a role of *Tg*AP1 in either of these pathways (Fig 11). Indeed, in mammalian cells, AP1 also regulates the retrograde transport of proteins from the early/sorting endosomal compartment to the TGN, while GGA (Golgi-localized, γ ear-containing, ADP-ribosylation factor-binding) is involved in anterograde transport from the TGN to the endosomes [27]. GGA molecules are not encoded in the *T*. *gondii* genome [11]. Thus, one can envision a similar role for *Tg*AP1 in the retrograde transport of molecules from the Rab5A-positive ELC to the TGN, in particular during rhoptry biogenesis. Indeed, rhoptry biogenesis could follow similar mechanisms than the ones involved in secretory granule maturation. In specialized secretory cells, immature secretory granules emerge from the TGN as preformed large vesicular compartments [36], similar to what we observed for the PreROP compartments in *T*. *gondii*. Mature granule formation requires an AP1-dependent retrieving of SNARES and proteases from the immature granule in order to form granules competent to be released upon an external stimulus. Therefore, it is possible that similarly, the defect in the preROP maturation process that we observed is the consequence of an impairment of *Tg*AP1-dependent retrieving of membrane factors, such as SNAREs. This mechanism would be crucial to pursue the rhoptry maturation process, in particular the further steps of transport, apical anchoring and final remodeling of the preROP compartments into club-shaped organelles. This hypothesis would be in line with data obtained in other eukaryotic systems showing that AP1 triggers clathrin-dependent vesicular budding from Rab5- and Rab4-positive early/sorting endosomal compartments [55] [28] [29]. We also observed that perturbing *Tg*AP1 function induces a more general defect in the Rab5A-positive compartment, distinct from immature pre-rhoptries, suggesting that a specific functional relationship might exist between *Tg*AP1 and the Rab5A-positive compartment in *T*. *gondii*. At the molecular level, AP1 and GGA1 proteins have been demonstrated to regulate Rab5 membrane dynamics by binding directly to the Rab5 effector Rabaptin-5 in mammalian cells [56] [57]. Notably, over-expression of Rabaptin-5 shifts the localization of GGA1- and TGN-associated cargos into enlarged Rab5 endosomes [56] [57] [58]. Therefore, further studies are needed to explore a putative role of *Tg*AP1 in the regulation of the Rab5 membrane dynamics in *T*. *gondii* and to identify the involved molecular mechanisms.

Moreover, the fact that a unique ENTH-domain containing protein is expressed in *T*. *gondii* raised the question of the role of clathrin-mediated endocytosis at the plasma membrane, a question that is still a matter of debate. In particular, clathrin was found mainly localized at the TGN and in cytoplasmic vesicles and perturbing its function led to defects in Golgi duplication and ROP and MIC biogenesis [59]. However, it was recently shown that parasites depleted for the cathepsin CPL internalized GFP proteins by a still unknown mechanism [60]. The role of clathrin- or *Tg*AP2-mediated endocytosis in this process was not investigated. Here, we found that the unique *T*. *gondii* epsin-like protein mostly co-localizes with *Tg*AP1 at the TGN and in cytoplasmic vesicles and that *Tg*EpsL interacts with the *Tg*AP1 complex but not the *Tg*AP2 complex. These findings argue against a clathrin- and epsin-mediated mechanism for protein internalization at the parasite plasma membrane, but further experiments are required to confirm this hypothesis. In particular, we are currently investigating *Tg*EpsL function by the generation of inducible KO parasites. Due to the unique organisation of the parasite cortex, that comprises three lipid bilayers (the plasma membrane and the inner membrane complex), it is possible that *T*. *gondii* uses alternative specific pathways for the internalization of macromolecules compared to mammalian cells.

### *Tg*AP1 regulates parasite growth

Our data strongly suggest that the cell division defect we found upon APμ1 ablation is not linked to rhoptry and microneme biogenesis but rather to a *Tg*AP1-mediated vesicular transport at the level of the basal pole and lateral sides of segregating daughter cells. Indeed, we could already observe at the stage of 4 parasites per vacuole, the ROP and MIC protein trafficking defects before the cell division process was drastically affected (Fig 4 and Fig 5). In APμ1-KO parasites, the budding process of nascent daughter cells was not perturbed as well as the formation of the MORN1-positive contractile rings. However, we observed both by IFA (Fig 8H) and electron microscopy (Fig 9), that the basal pole of the parasites is elongated and deformed, suggesting a later defect after the contractile rings of the basal complex have reached the posterior end of the parasite. In higher eukaryotes, mid-body abscission, which takes place last at the end of cytokinesis, is a complex process timely regulated by the sequential recruitment of different factors such as members of the ESCRT protein family, the Chromosomal Passenger Complex, Rab GTPAses and kinase/phosphatases [51]. *In T*. *gondii*, after daughter cell budding has been completed, parasites remained attached within the vacuole by their basal pole via a highly organized structure called the residual body [61]. The role of the residual body is not clear. Apart from receiving the remnant material from the mother parasite, it probably plays an important role in the maintenance of synchronous division cycles within the vacuole. A recent study has described the well organized and regular structure of the residual body, connecting each parasite basal pole via a thin membranous connection, which follows the constricted basal complex region [61]. This organization seems to be perturbed in *Tg*AP1–defective parasites. Thus, two scenarios could be proposed to explain the division defects observed in APμ1-KO parasites. First, a *Tg*AP1-dependent delivery of vesicles directly from the TGN/ELC to the basal pole could be required to orchestrate the final step of cytokinesis and in particular, to spatially organise the specific attachment structure that remains between parasites. These vesicles could deliver crucial regulatory factors, such as regulators of cytoskeleton components required to modulate the contractile force involved in parasite attachment at the residual body. Second, *Tg*AP1 could be involved in a recycling activity of the mother plasma membrane from the residual body to terminate daughter cell segregation, such as previously observed for the IMC [62] or contribute to a direct transport of *de novo* synthesized lipids from the TGN to the plasma membrane. In agreement with the latter hypothesis, a recent study has demonstrated that parasite depleted for the FAS II enzyme, which is responsible for fatty acid biosynthesis at the apicoplast, displayed drastic division defects and were unable to segregate after the budding process has completed, forming a mass of tethered cells [63]. This study revealed the requirement for *de novo* lipid synthesis and therefore, we believe, of regulated trafficking pathways for the delivery of these lipids, to complete daughter cell segregation at the end of cytokinesis. Importantly, a similar role for the AP1-dependent delivery of Golgi-derived vesicles at the cleavage furrow of dividing cells, has been previously described in different organisms, such as *S*. *pombe* [41], *D*. *discoideum* [42], *C*. *elegans* embryo and plants [32], suggesting a conserved function for the complex AP1 in cell division among various eukaryotic organisms including *T*. *gondii*. Therefore, a major perspective of our work will be to dissect the mechanisms regulating a possible *Tg*AP1-mediated transport of vesicles from the parasite basal pole and/or the TGN/ELC to the cortical area and to identify cargos that could be transported via this pathway and possible regulatory factors. In particular, it will be interesting to investigate potential links with other compartments involved in constitutive secretion such as the Rab11A-positive compartment. Indeed, deregulation of Rab11A activity also results in incomplete pellicle assembly in the inner regions between daughter cells, leading to a cell separation block late in cytokinesis [53].

In conclusion, in plant cells, secretory and endocytic routes intersect at the hybrid trans-Golgi network /early endosomes, where cargos from both the anterograde and the retrograde pathways are further correctly sorted in a timely manner [44]. In *T*. *gondii*, we also found a tight physical and functional association between the TGN and the ELC throughout the cell cycle. BFA treatment led to the dispersion of both, the TGN and the Rab5A-positive compartment. Although tightly connected, these two compartments are functionally distinct, as recently suggested by the study of the retromer functions, where depletion of *Tg*Vps35 led to the retention of SORTLR in the endosomal-like compartment by inhibition of its retrograde transport to the TGN [26]. In plants, late endosomes, also called multi-vesicular bodies (MVBs) or pre-vacuolar compartments (PVCs) were shown to directly emerge from the hydrid TGN/early endosomal compartment as immature large vesicular compartments containing intra-luminal membranes, such as observed for the preROP compartments in *T*. *gondii* [64]. Furthermore, AP1 was shown to be critical for BFA-sensitive post-Golgi trafficking events from the TGN/EE to the MVB [30] [32]. Finally, fully differentiated APμ1-KO plant cells contained fragmented vacuoles rather than a large central vacuole as in wild type cells, suggesting an additional role of AP1 in the later step of vacuolar fusion similar to what is observed in yeast [32] [33]. Our findings suggest that AP1 in *T*. *gondii* may also be involved in both steps of proROP protein exit from the TGN compartment towards the ELC but also at the later step of preROP vesicular compartments maturation into apically anchored club-shaped rhoptries. Because of the functional and structural similarities between *T*. *gondii* and the plant trafficking system, one can envision that many vesicular trafficking pathways and the corresponding molecular regulatory mechanisms are conserved.

## Materials and methods

### Ethics statement

No study on human participants, specimens or tissue samples, or vertebrate animals, embryos or tissues have been conducted.

### Cloning strategies

Genomic DNA was isolated from the Type I RHΔKU80 strain parasites using the Promega Wizard genomic DNA purification kit and used as template for PCR. The p5RT70 loxp-AP1μ-loxp-YFP-HXGPRTplasmid was generated by a 3 step cloning. First, a 2kb fragment from the endogenous 3’UTR of *Tg*APμ1 (TGGT1_289770) was amplified using primers CCGGGAGCTCAAAATCAACAAGGGGGGGCGAGG and GCGCGAGCTCACGGAGAAGGAACGAGGAGCAAAG and cloned into a unique SacI site of the mother vector p5RT70loxPKillerRedloxPYFP-HXGPRT [48]. As a second step the coding sequence of the gene was amplified with a HA epitope tag added at the C terminus using primers GCGCCCTAGGATGGCGGGGGCGTCTGCGGTGT and GCGCAGATCTCTAAGCGTAATCTGGAACATCGTATGGGTAGGAGAGTCTCAGTTGGTACTCTCCA and inserted into the plasmid using the restriction sites AvrII and BglII respectively. As a final step, a 2.5kb fragment from the 5’UTR of *Tg*APμ1 was amplified with the primers GCGCGGTACCCAAGTTCCCGTTTGTCCTGG and GCGCGGGCCCTCTTGGGACTGCAAGATCGACTG cloned using the sites KpnI and ApaI respectively. The DDcMycμ1 parasites were obtained using the ddFKBP over-expression system [49] as follows: The *Tg*APμ1 gene was amplified with the following primers: GCGCATGCATATGGCGGGGGCGTCTGCG and GCGCTTAATTAACTAGGAGAGTCTCAGTTGGTACTCTCCATTTTGAGTGATG and cloned into the pG12-Tub8-DD-mCherrycMyc-HXGPRT vector using the restriction sites NsiI and PacI. The plasmid was then digested by AvrII and BglII to remove the DD and mCherry fragments. The DD cassette was re-introduced into the resulting vector after amplification by PCR (F: CTTTTAGATCTAAAATGGGAGTGCAGG, R: GCGCCCTAGGTTCCGGTTTTAGAAGCTCCAC) and ligation into the AvrII and BglII sites.

Primers used to generate 3’-terminally tagged genes integrated at the endogenous locus (knock-in parasites) and produce recombinant proteins are indicated in Table 4.

**Table 4:** 

Plasmid	Primers (F: Forward; R: reverse)	Linearization enzyme
pLic EpsL-cmyc (HXGPRT)	F: TACTTCCAATCCAATTTAATGCCCTCGTTCTCTCCTTCTCAGACGTT	NcoI
	R: TCCTCCACTTCCAATTTTAGCGAACCCCGTCGTAGCAGGAGAT	
pLic μ1-HA (DHFR)	F: TACTTCCAATCCAATTTAATGCGGATCTTCCCTAGTTCGCGCCAGTCAC	SnaBI
	R:TCCTCCACTTCCAATTTTAGCGGAGAGTCTCAGTTGGTACTACTCTCCATTTTGAGT	
pLic-Rab5A-YFP (DHFR)	F: TACTTCCAATCCAATTTAATGCACTTTTGCCTCCACATGCACACC	Eco47III
	R: TCCTCCACTTCCAATTTTAGCGTGAGTGTCTCAGAAGGGAAGAACG	
pLic σ1-HA (DHFR)	F: TACTTCCAATCCAATTTAATGCGTGATCCACCACTTTGTCGAGATCTTGG	EcoRV
	R: TCCTCCACTTCCAATTTTAGCGTCATGTAAGCTTGACTCCACCTTTAGTGTTGCTC	
GST-βear	F: GGATCCGAGAACTCCTCTGCCGACAAGGACGTTTTCAGA	
	R: GAATTCTCACGACCGTGGCGTCAGCC	
GST-γear	F: GGATCCTTTCCGCCGATGAATGTCTTGAACGAGGACG	
	R: GAATTCTCACGCGAGGAGTCCCGCGG	

### Parasite culture and transfection

*Toxoplasma gondii* Type I RHΔKU80ΔHXGPRT and DiCreΔKU80ΔHXGPRT parasites were grown on confluent Human Foreskin Fibroblast (HFF) cells (CCD-1112Sk (ATCC, CRL-2429^TM^)) which were cultured in complete DMEM (gibcoLife Technologies) supplemented with 10% Fetal Bovine Serum (GibcoLife Technologies) and 1% Pen Strep (gibcoLife Technologies). To obtain the DDμ1 parasites, 50μg of the pG12-Tub8-DD-cmyc-APμ1-HXGPRT plasmid was transfected in RHΔKU80ΔHXGPRT parental strain by electroporation following standard procedures. To obtain the APμ1-KO parasites, 50μg of loxp-APμ1HA-loxp-YFP-HXGPRT construct was transfected in the DiCreΔKu80ΔHXGPRT strain parasites. Following transfection, in both cases the parasites were subjected to Mycophenolic acid/Xanthine drug selection and verified for the transfection efficiency by immunofluorescence analysis. Subsequently the non-clonal populations of parasites were subjected to cloning by serial dilution. For the APμ1-KO clonal parasites, integration of the transgenic construct at the endogenous locus was verified by a genotyping PCR using a forward primer (GACGCGTTTCACTTCCTCTGCTTCCTC) located upstream of the cloned 5’UTR, and a reverse primer (GTTTACGTCGCCGTCCAGCTCGAC) located on the YFP cassette. To obtain clonal knock-in parasites, 25 μg of plasmids were linearized over-night and transfected into the RHΔKU80ΔHXGPRT parental strain by electroporation followed by drug selection and cloning. Transient transfections were performed in 10*10^6^ parasites with 50 μg of the following plasmids: HA–tagged *Tg*Rab5A (V. Carruthers) / cMyc–tagged *Tg*Rab7 and cMyc–tagged *Tg*Rab5A (M. Meissner); GalNac-YFP (D. Roos); GRASP-RFP (K. Hager), MORN1-cherry and IMC3-cherry (M.J. Gubbels) and parasites were allowed to invade HFF cells for 24 h prior analysis.

### Western blot

Parasites were lysed in lysis buffer (NaCl 150mM, TrisHCl 20mM, EDTA 1mM, 1% TritonX100, protease inhibitors) and total proteins were subjected to electrophoresis in a 10% polyacrylamide gel. The proteins were transferred onto a nitrocellulose membrane (Amersham^TM^Protran^TM^ 0.45μ NC) by a standard western blot procedure. The membrane was blocked with 5% milk (non-fat milk powder dissolved in TNT buffer: 100mM Tris pH8.0, 150mM NaCl and 0.1% Tween20) and probed with primary antibodies diluted in the blocking buffer. The primary antibodies were followed by respective species specific secondary antibodies conjugated to HRP. The antibody incubations were followed by thorough washing using the TNT buffer. The membranes were visualized using ECL Western blotting substrate (Pierce).

### Immunofluorescence assays (IFA)

When indicated, infected confluent HFF monolayers were incubated for 1 h with 5 μM of Brefeldin A (Sigma-Aldrich) before fixation with 4% paraformaldehyde (PFA) in phosphate buffered saline (PBS), for 20 minutes. After quenching with 50mM NH_4_Cl, the coverslips were permeabilised with 0.2% triton dissolved in 5% FBS-PBS for 30 minutes. The coverslips were then incubated with primary antibodies in 0.1% triton dissolved in 2%FBS-PBS or 0.05% Saponin for 1 h and then washed with PBS, followed by goat anti-rabbit or goat anti-mouse secondary antibodies conjugated to Alexa Fluor 488 or Alexa Fluor 594 (Molecular Probes, Invitrogen). Images were acquired using a Zeiss LSM880 confocal microscope. Antibodies used for IFA experiments are the following: rabbit anti-HA (Cell Signaling Technology), rat anti-cMyc (Abcam), mouse anti-SAG1 (our lab), rabbit anti-GAP45, rabbit anti-MIC8, anti-MIC4, anti-MIC6 (D. Soldati-Favre), mouse anti-MIC2, rabbit anti-M2AP, mouse anti-proMIC3, rabbit anti-proM2AP (V. Carruthers), mouse anti-MIC3, mouse anti-ROP 2–4, mouse anti-GRA3 (J.F. Dubremetz), rat anti-SORTLR (our lab), mouse anti-chromo1 (our lab), rabbit anti-proROP4 (G.E Ward), mouse anti-IMC1 (M.J. Gubbels) and rabbit anti-GRA6 (C. Mercier). To quantify the percentage of vacuoles presenting apically positioned rhoptries and proROP4-positive vacuoles in control parasites or induced APμ1-KO and DDμ1 parasites, a total of 150 vacuoles were monitored for each condition in 3 independent assays. Data are presented as mean ± Standard Error Mean (SEM).

### Structured illumination microscopy (SIM)

SIM was used to obtain high-resolution images using an ElyraPS1 microscope system (Zeiss) with a 100x oil-immersion lens (alpha Plan Apochromat 100x, NA 1.46, oil immersion) and a resolution of 120 nm along the x-y axis and 500 nm along the z-axis (PSF measured on 100 nm beads; Sampling voxel size: 0,050μm*0,050μm*0,150 μm). Three lasers (405, 488, and 561 nm) were used for excitation. SIM images were acquired with an EMCCD camera (Andor Technology Ltd, UK) and processed with ZEN software, where exposure times varied between 100 and 150 ms. Three-dimensional images were generated using a z-step of 150 nm (total thickness ~5 μm). The acquisition was done sequentially using Zeiss Filter Sets 43HE, 38HE and BP 420–480. 15 frames were acquired to reconstruct one image (5 rotations x 3 phases, with a SIM Grating period of 51μm for the blue channel, 42 μm for the green channel, 34μm for the red channel). 100 nm beads were imaged to measure the chromatic mis-alignment of our system (fit procedure by the Zen software); this parameter enabled correcting the alignment on each acquired multi-channel stack. Image reconstructions and co-localization quantification were determined with IMARIS software (Bitplane).

### Intracellular growth assay

APμ1-KO parasites were allowed to invade HFF monolayers for 3 h and treated with 50nM Rapamycin for 6 h. After 3 washes with warm medium, parasites were allowed to grow for additional 16 h before fixation with 4% PFA. For the DDμ1 strain, parasites were inoculated onto HFF monolayers for 3 h and treated with or without shield-1 (1μM) for 16 h, before fixation with 4% PFA. In both cases, intracellular parasites were counted after staining with anti-GAP45 antibodies. The numbers of parasites per vacuole were counted for more than 200 vacuoles for each condition performed in duplicate. Data are presented as mean values of three independent assays ± SEM.

### Invasion assay

Intracellular DDμ1 transfected parasites induced with or without 1μM Shield for 16 h or intracellular APμ1-KO parasites induced with 50 nM rapamycin for 6 hours and allowed to grow for an additional 16 hours were mechanically released from host HFF cells. Two million parasites were then allowed to adhere to host cell monolayers by centrifugation for 3 min at 1200rpm then shifted to 37°C for 45 min. Non adherent parasites were washed away with PBS followed by fixation with 4% PFA for 10min. The red-green invasion staining procedure was followed as described earlier [49]. Briefly, adherent external parasites were labeled with mouse anti-SAG1 antibodies, followed by secondary anti-mouse antibodies coupled to Alexa594. After cell permeabilisation with Triton 0.1% for 10 min, invaded intracellular parasites were detected using rabbit anti-GAP45 antibodies followed by secondary anti-rabbit antibodies coupled to Alexa488. For APμ1-KO parasites, YFP-positive parasites were counted for their invasion capacity compared to non-YFP neighbouring parasites present on the same coverslips. At least, 300 parasites (for DDμ1 and control parasites) and 150 parasites (APμ1-KO) were counted for each condition. Data are presented as mean values of three independent assays ± SEM.

### Egress assay

Host cells (HFFs) were seeded in 8-well chambers (Nunc® Lab-Tek® II chambered coverglass). 5*10^4^ freshly egressed parasites per well were seeded onto HFF monolayers and allowed to invade for 2 hours. Parasites were then treated with 50nM Rapamycin for six hours. Subsequently medium was changed and parasites were allowed to grow further for 24 hours. The chamber was then placed on an inverted microscope (Axio-observer, Zeiss) equipped with an incubation chamber set at 37°C, and supplied with 5% CO2. Egress was induced with 2μM calcium ionophore A23187 (Sigma-Aldrich).The movies were captured using a 40X Plan apochromat NA 1.4 objective. Image acquisition was performed using AxioVision Software (Zeiss) for up to 10 minutes on each well. A total of 50 YFP-positive and 150 YFP-negative vacuoles were monitored. Data are presented as mean values of three independent assays ± SEM.

### Immunoprecipitation

For immunoprecipitation assays, a minimum of 0.6 billion parasites of APμ1-HA, EpsL-cMyc / pLIC-APμ1-HA, and DDμ1 strains were lysed on ice for 30 min in modified RIPA buffer (50mM TrisHCl pH8.0, 2mM EDTA, 75mM NaCl, 0.65% NP40, 0.005%SDS, 0.5mM PMSF) and centrifuged at 14 000 rpm for 15 min to remove cell debris. Protein concentration was determined using the BCA protein assay kit (Pierce^TM^). 500μg of total lysate were immunoprecipitated by binding to 50μl of anti-cMyc agarose beads (Pierce^TM^) or anti-HA agarose beads (Pierce^TM^) overnight. After five washes of 10 min each with modified RIPA buffer, bound proteins were eluted by boiling the samples in laemmeli buffer. Samples were then subjected to SDS PAGE and western blotting or gel-extracted for tryptic digestion and mass spectrometry analysis.

### GST pull-down

The C-terminal ear appendage domain of the β (BAE) and γ (GAE) subunits were GST tagged by cloning into a pGEX6p3 vector (Pharmacia). Expression of GST-BAE and GST-GAE in BL21 competent cells was achieved by induction with 1mM IPTG at 37°C for 4 h. Bacteria lysates expressing GST-BAE, GST-GAE, and GST (control) were bound to 100μl of Protino Glutathione agarose 4B beads (Machery Nagel) in GST-lysis/binding buffer (Tris HCl (pH 7.6) 50mM EDTA 1mM, EGTA 1mM, 2-mercaptoethanol 10mM, NaCl 150mM, TritonX-100 0.5%, and PMSF 0.5mM) overnight at 4°C. The beads were washed 5 times with wash buffer A (Tris HCl (pH 7.6) 50mM, 2-mercaptoethanol 10mM, NaCl 500mM, Triton 0.5% and PMSF 0.5mM) and 3 times with wash buffer B (Tris HCl (pH 7.6) 20mM, NaCl 150mM, NP40 0.65%, SDS 0.005%, PMSF 0.5mM) sequentially. Beads containing 150μg of the recombinant proteins and the control GST protein were incubated with a lysate from 0.4 billion EpsL-cMyc / μ1-HA intracellular parasites overnight at 4°C. Parasites were lysed using modified RIPA (TrisHCl (pH8.0) 50mM, EDTA 2mM, NaCl 75mM, NP40 0.65%, SDS 0.005%, PMSF 0.5mM). After 3 washes with the lysis buffer, the proteins bound to the beads were eluted with 1x Laemelli blue buffer by boiling. The samples were subject to western blot and mass spectrometric analyses.

### Mass spectrometry proteomic analysis

After denaturation at 100°C in 5% SDS, 5% βmercaptoethanol, 1 mM EDTA, 10% glycerol, 10 mM Tris buffer pH 8 for 3 min, protein samples were fractionated on a 10% acrylamide SDS-PAGE gel. The electrophoretic migration was stopped as soon as the protein sample entered 1 cm into the separating gel. The gel was briefly stained with Coomassie Blue, and five bands, containing the whole sample, was cut. In gel digestion of gel slices was performed as previously described [65]. An UltiMate 3000 RSLCnano System (Thermo Fisher Scientific) was used for separation of the protein digests. Peptides were automatically fractionated onto a commercial C18 reversed phase column (75 μm×150 mm, 2 μm particle, PepMap100 RSLC column, Thermo Fisher Scientific, temperature 35°C). Trapping was performed during 4 min at 5 μl/min, with solvent A (98% H2O, 2% ACN and 0.1% FA). Elution was performed using two solvents A (0,1% FA in water) and B (0,1% FA in ACN) at a flow rate of 300 nl/min. Gradient separation was 3 min at 5% B, 37 min from 5% B to 30% B, 5 min to 80% B, and maintained for 5 min. The column was equilibrated for 10 min with 5% buffer B prior to the next sample analysis. The eluted peptides from the C18 column were analyzed by Q-Exactive instruments (Thermo Fisher Scientific). The electrospray voltage was set at 1.9 kV, and the capillary temperature was set at 275°C. Full MS scans were acquired in the Orbitrap mass analyzer over m/z 300–1200 range with resolution 35,000 (m/z 200). The target value was 5.00E+05. Ten most intense peaks with charge state between 2 and 4 were fragmented in the HCD collision cell with normalized collision energy of 27%, and tandem mass spectrum was acquired in the Orbitrap mass analyzer with resolution 17,500 at m/z 200. The target value was 1.00E+05. The ion selection threshold was 5.0E+04 counts, and the maximum allowed ion accumulation times were 250 ms for full MS scans and 100 ms for tandem mass spectrum. Dynamic exclusion was set to 30 s.

### Proteomic data analysis

Raw data collected during nanoLC-MS/MS analyses were processed and converted into *.mgf peak list format with Proteome Discoverer 1.4 (Thermo Fisher Scientific). MS/MS data was interpreted using search engine Mascot (version 2.4.0, Matrix Science, London, UK) installed on a local server. Searches were performed with a tolerance on mass measurement of 0.2 Da for precursor and 0.2 Da for fragment ions, against a composite targetdecoy database (50620 total entries) built with 3 strains of *Toxoplasma gondii* ToxoDB.org database (strains ME49, GT1 and VEG, release 12.0, September 2014, 25264 entries) fused with the sequences of recombinant trypsin and a list of classical contaminants (46 entries). Cysteine carbamidomethylation, methionine oxidation, protein N-terminal acetylation and cysteine propionamidation were searched as variable modifications. Up to one trypsin missed cleavage was allowed.

### Correlative light electron microscopy (CLEM)

Host cells were cultured on alphanumerical gridded-glass bottom dishes (P35G-1.5-14-CGRD, MatTek Corporation, Ashland, MA, USA) until 50% confluence was reached. Parasites were allowed to invade for 2 h, washed twice with warm medium, then induced for 6 h with Rapamycin 50n M, washed thrice with PBS and allowed to grow for additional 16hrs. Cells were then fixed with 4% PFA / 0.5% glutaraldehyde in PBS over-night. YFP-positive *Tg*APμ1-KO parasites were imaged using a Zeiss LSM880 confocal microscopy and localized on the alphanumerical grid using transmitted light. After observation, cells were fixed with 2% glutaraldehyde in 0.1 M sodium cacodylate buffer over-night. After washing with water, cells were sequentially stained with 1% osmium tetroxide reduced with 1.5% potassium hexacyanoferrate(III) for 1 hour, 1% thiocarbohydrazide for 30 minutes, 1% osmium tetroxide, 1% uranyl acetate overnight at 4°C, and finally lead aspartate for 3 h. All stains were made in water, in the dark and at room temperature unless otherwise indicated. All stains were also washed with water. After staining, cells were dehydrated in graded ethanol solutions, infiltrated with epoxy resin and cured at 60°C for 48 h. After separation of the resin from the glass, cells of interest were relocated with the imprinted-alphanumerical grid at the surface of the resin. Small blocks of resin containing the cells of interest were prepared for sectioning parallel to the resin surface. Serial sections of 80 nm thickness were set down on carbon/formvar-coated slot grids. Sections were observed with a Hitachi H7500 TEM (Elexience, France), and images were acquired with a 1 Mpixel digital camera from AMT (Elexience, France).

### Transmission electron microscopy

After infection of a confluent HFF monolayer, cells containing replicating shield-1 induced DDμ1 or control parasites were detached with a scraper, spun down and fixed with 1% glutaraldehyde in 0.1 M sodium cacodylate pH 6.8 overnight at 4°C. Cells were post-fixed with 1% osmium tetroxide and 1.5% potassium ferricyanide for 1 hour, then with 1% uranyl acetate for 45 minutes, both in distilled water at room temperature in the dark. After washing, cells were dehydrated in graded ethanol solutions then finally infiltrated with epoxy resin and cured for 48 hours at 60°C. Sections of 70–80 nm thickness on formvar-coated grids were observed with a Hitachi H7500 TEM (Elexience, France), and images were acquired with a 1 Mpixel digital camera from AMT (Elexience, France).

### Statistics

Means and SEM and SD were calculated in Excel. *P*-values were calculated in Excel using the Student’s *t*-test assuming equal variance, unpaired samples and using two-tailed distribution.

## Supporting information

S1 Fig **A-** Confocal microscopy images showing the co-localization of μ1-HA (green) and cMyc-tagged Rab5A (red) at the duplicated Golgi during the G1/S phase of the cell cycle in μ1-HA KI parasite. Bar: 2 μm. **B- μ**1-HA KI parasites (upper panel, green) or RH parasites transiently transfected with cMyc-tagged Rab5A (lower panel, green) were treated (or not) with Brefeldin A for 1 h before fixation and processing for IFA. SORTLR was used as a marker for the TGN compartment (red). The images show the dispersion of μ1-HA and Rab5A in vesicles and aggregates also positive for SORTLR. Bars: 2 μm.
(TIF)

S2 Fig (Supplementary data for Table 1). Detailed list of proteins identified by mass spectrometry after immunoprecipitation of the μ1-HA subunit using anti-HA antibodies in KI parasites expressing μ1-HA under the endogenous promotor. The parental strain RHΔKU80 has been used as a control for non-specific binding on the anti-HA antibody coated beads.
(XLSX)

S3 Fig **A**- WB image showing the expression of the HA-tagged σ1 subunit of *Tg*AP1 at the expected size (20 kDa) in KI parasites and its absence in the parental RHΔKU80 strain using anti-HA antibody. **B**- SIM images showing the localization of σ1-HA (green) at the TGN together with SORTLR (red). The white frame indicates the region zoomed in and shown as insets in the lower panel of images. Bar: 2 μm. **C-** Sequence alignment of the unique *T*. *gondii* ENTH-domain containing protein (TGGT1_214180) with *Human* epsin1 (*Hs*, UniProtKB: Q9Y6I3) and epsinR (CLINT1/Epsin4, UniProtKB: Q14677), with *Arabidopsis thaliana* epsinR2 (*At*, UniProtKB: Q67YI9) and *Plasmodium falciparum* (*Pf*) unique ENTH-domain containing protein (PF3D7_1245800). A scheme illustrating the positions of the identified conserved domains in *Tg*EpsL, such as the ENTH domain, the clathrin binding site (DLL/LXD) and the NPF motif (the latter being predicted to mediate the association of epsin proteins with clathrin adaptor complexes), is shown below.
(TIF)

S4 Fig (Supplementary data for Table 2). Detailed list of proteins identified by mass spectrometry following the IP of EpsL-cMyc protein in double KI parasites expressing EpsL-cmyc and μ1-HA proteins. The single KI parasites expressing μ1-HA was used as a control for non-specific binding to the anti-cMyc antibody-coated beads.
(XLSX)

S5 Fig The schemes illustrate the conserved identified domains of the AP1-γ (TGGT1_240870) and AP1-β (TGGT1_313670) subunits of the AP1 complex, including the N-terminal Adaptin domain, the clathrin binding sites present in the hinge domain and the C-terminal appendage ear (AE) domains. Sequence alignments of the GAE and BAE domains of *T*. *gondii* AP1 (*Tg*) with the corresponding sequences found in the AP1 complex of *Plasmodium falciparum* (*Pf*), *P*. *berghei* (*Pb*), *Arabidopsis thaliana* (*At*) and humans (Hs) are also shown. The accession numbers of analyzed proteins are indicated in the S1 Table.
(TIF)

S6 Fig **A, B-** Confocal images showing the localization of AMA1 (A), MIC1 (A) and SORTLR (B) proteins (in red) in control (YFP-negative) and APμ1-KO parasites (YFP-positive). SORTLR was not found mis-localized in APμ1-KO. **C-** Confocal images showing the localization of GRA3 (green) and GRA6 (red) in YFP-positive APμ1-KO parasites (yellow). No defect in dense granule biogenesis was observed upon APμ1 depletion. All bars: 2 μm.
(TIF)

S7 Fig **A-** Confocal images showing the endogenous localization of MIC2, M2AP, MIC3 and MIC8 (all in green) in micronemes of DDμ1 parasites over-expressing the cMycμ1 protein (red) after shield-1 induction for 24 hours (+S). Bars: 2 μm. **B**- WB image showing no differences in the expression and proteolytic maturation of MIC2, MIC8, MIC3 and M2AP proteins in shield-1 induced control parasites (left) and DDμ1 parasites (right) for 24 h. Actin (ACT1) was used as loading control. **C-** Confocal images showing the co-localization of SORTLR (white) with proROP4 (green) and cMycμ1 (red) in control (RH) and DDμ1 parasites incubated with Shield-1 for 24 h. A zoom of the Golgi region indicated by a white frame on the merged image is shown as inset. Bars: 2 μm. **D-** Histogram indicating the percentage of co-localization between the proROP4 signal and the SORTLR signal after image acquisition by airyscan microscopy. Data are indicated as average ± SD, n≥15 vacuoles (Student’s t-test). **E**. Confocal images displaying the localization of the endogenous σ1-HA protein (green) in cMycμ1-overexpressing parasites (red) induced with shield-1 for 16 hours. The endogenous σ1-HA subunit was found localized at the Golgi together with the cMycμ1 protein. Bars: 2 μm.
(TIF)

S8 Fig (Supplementary data for Table 3). Detailed list of proteins identified following immunoprecipitation of the over-expressed cMycμ1 in DDμ1-induced parasites using anti-cMyc antibodies. The parental strain RH was used as a control for non-specific binding on the anti-cMyc antibody coated beads.
(XLSX)

S9 Fig Confocal images showing daughter cell bud formation revealed by a tubulin staining (green, upper panel, arrows) and centromere duplication labeled with the protein chromo1 (green, lower panel, arrows) in shield-1 induced DDμ1 parasites (cMycμ1-overexpressed protein in red). A zoom of the region indicated by a white frame in the merge image is shown in the inset on the right. Note the accumulation of the cMycμ1 protein at the basal pole of connected parasites. Bars: 2μm.
(TIF)

S10 Fig **A- upper panel:** strategy used to perform CLEM microscopy. HFF cells were allowed to grow (50% confluent) on alphanumerical coverslips. Confocal microscopy images were taken (40X objective) to spot the YFP positive parasites corresponding to APμ1-KO parasites or control non-YFP vacuoles. A mosaic of 8*8 microscopy fields centred on the parasites of interest was then acquired to determine the vacuole position on the grid as illustrated for the region 3T, corresponding to the APμ1-KO vacuole shown in Fig 9. The YFP-positive vacuoles at the positions 6H and 3K (arrows) correspond to vacuoles analyzed by CLEM and shown in **B** (image c: 6H and images d, e, f: 3K). **B-** Typical mature rhoptries and apical / lateral micronemes were detected in non-YFP control parasites (images a and b, arrows). By contrast, in YFP-positive APμ1-KO, no mature rhoptries were visualized at the apical pole, which instead displayed numerous big vesicles and only apical micronemes were detected (images c and d, arrows). Drastically affected APμ1-KO vacuoles showed abnormal division, with parasites that have broken out or not fully segregated (image e). Image f corresponds to a zoomed region of image e (white frame) showing incomplete pellicle formation and separation between two neighbouring cells (arrows). Rh: rhoptries, MIC: micronemes, DG: dense granules. Bars: 500nm.
(TIF)

S11 Fig Transmission electron microscopy images showing the formation of enlarged lucent vesicles upon over-expression of APμ1. In control parasites (A-D), lucent vesicles were not often visualized or detected as small vesicles resembling endosomes (C and D, arrows). C represents a zoom of the region indicated by a white frame in B. In opposite, in induced DDμ1 parasites (E-L), large lucent vesicles accumulated mainly at the post-nuclear anterior region of the parasite (arrows). We often detected membranous or vesicle-like material within their lumen (E-F and I-J, arrow heads). In some cases, this material seems to be generated by internal budding of the limiting membrane (J, arrow heads). F, H and J represent a zoom of the region indicated by a white frame in E, G, and I, respectively. Bars: 100 nm or 500 nm as indicated.
(TIF)

S1 Table Accession numbers of the genes used for the sequence alignment analysis showed in S5 Fig of the Appendage Ear domain of the Gamma subunit (GAE) and the Beta subunit (BAE). (PDF)

S1 Movie 3D-SIM image reconstruction showing the localization of the Rab5A compartment (green) compared to APμ1 (red) localized in the TGN area. The KI parasite line expressing Rab5-YFP under the endogenous promoter was used for the study.
(AVI)

S2 Movie 3D-SIM image reconstruction showing the localization of the Rab5A compartment (green) compared to the TGN marker SORTLR (red). The KI parasite line expressing Rab5-YFP under the endogenous promoter was used for the study.
(AVI)

S3 Movie Time-lapse movie (one frame every 10 min for a duration of 5 hours) showing MORN1-cherry localization during the budding process in rapamycin induced APμ1-KO parasites (YFP-positive). Note that the basal complex is normally assembled and contractile rings developed towards the basal pole of budding daughter cells. Movies were captured using an inverted microscope (Axio-observer, Zeiss) equipped with a 40X Plan apochromat NA 1.4 objective. Image acquisition was performed using AxioVision Software (Zeiss).
(AVI)

S4 Movie Egress was triggered in rapamycin induced APμ1-KO parasites (YFP-positive) after incubation with the calcium ionophore A23187. Time-lapse movies were recorded for 10 minutes. While control parasites immediately initiated vacuole egress, some APμ1-KO parasites appeared tethered and were unable to escape the vacuolar space though vacuole lysis has occured.
(AVI)
