# Supplementary figures and images for: Dual role of the Toxoplasma gondii clathrin adaptor AP1 in the sorting of rhoptry and microneme proteins and in parasite division
Source: PLoS Pathog. 2017 Apr 21;13(4):e1006331. doi: 10.1371/journal.ppat.1006331 (PMC5415223; doi:10.1371/journal.ppat.1006331)

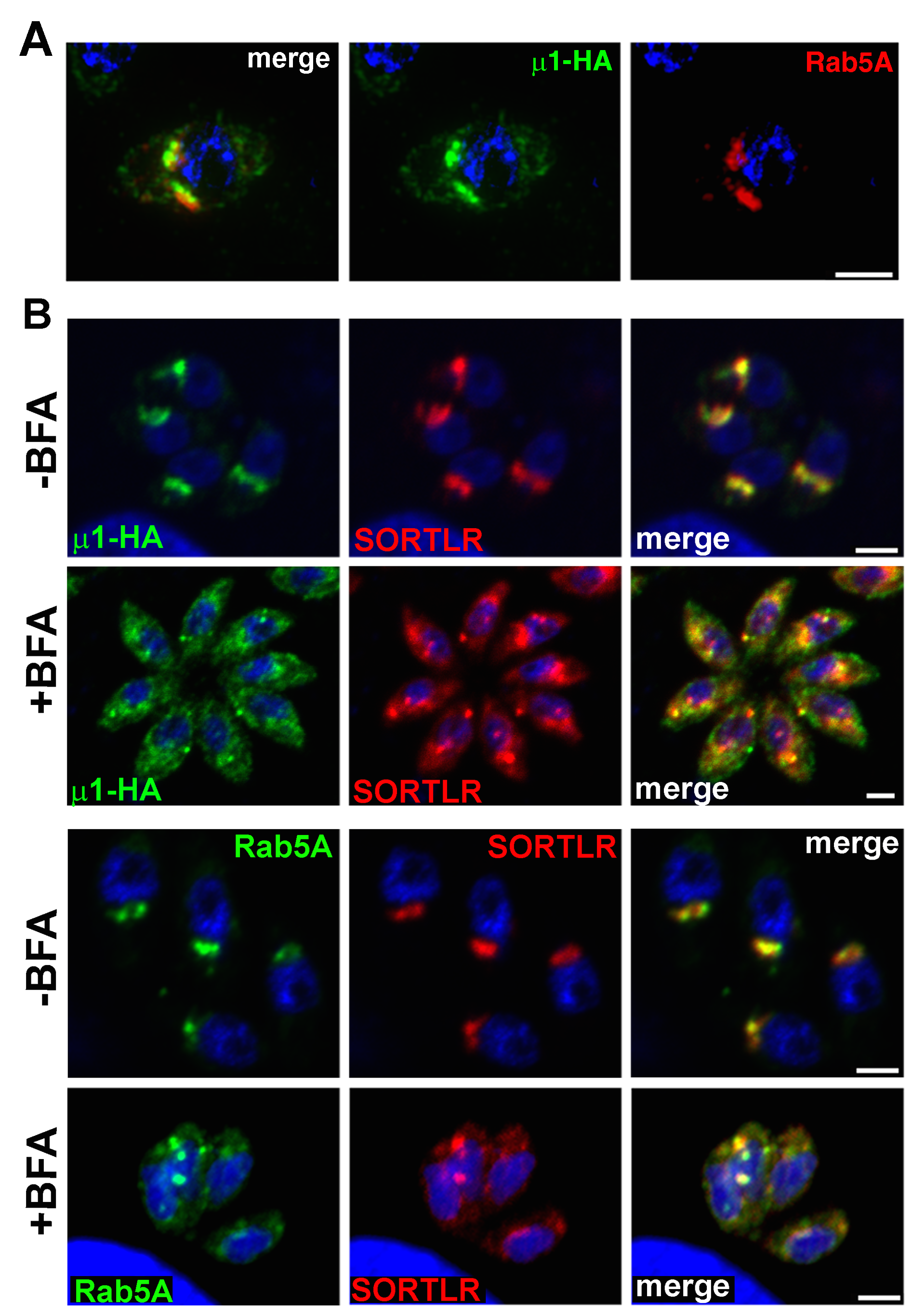

Supplement: S1 Fig — A- Confocal microscopy images showing the co-localization of μ1-HA (green) and cMyc-tagged Rab5A (red) at the duplicated Golgi during the G1/S phase of the cell cycle in μ1-HA KI parasite. Bar: 2 μm. B- μ1-HA KI parasites (upper panel, green) or RH parasites transiently transfected with cMyc-tagged Rab5A (lower panel, green) were treated (or not) with Brefeldin A for 1 h before fixation and processing for IFA. SORTLR was used as a marker for the TGN compartment (red). The images show the dispersion of μ1-HA and Rab5A in vesicles and aggregates also positive for SORTLR. Bars: 2 μm. (TIF) [file ppat.1006331.s001.tif]

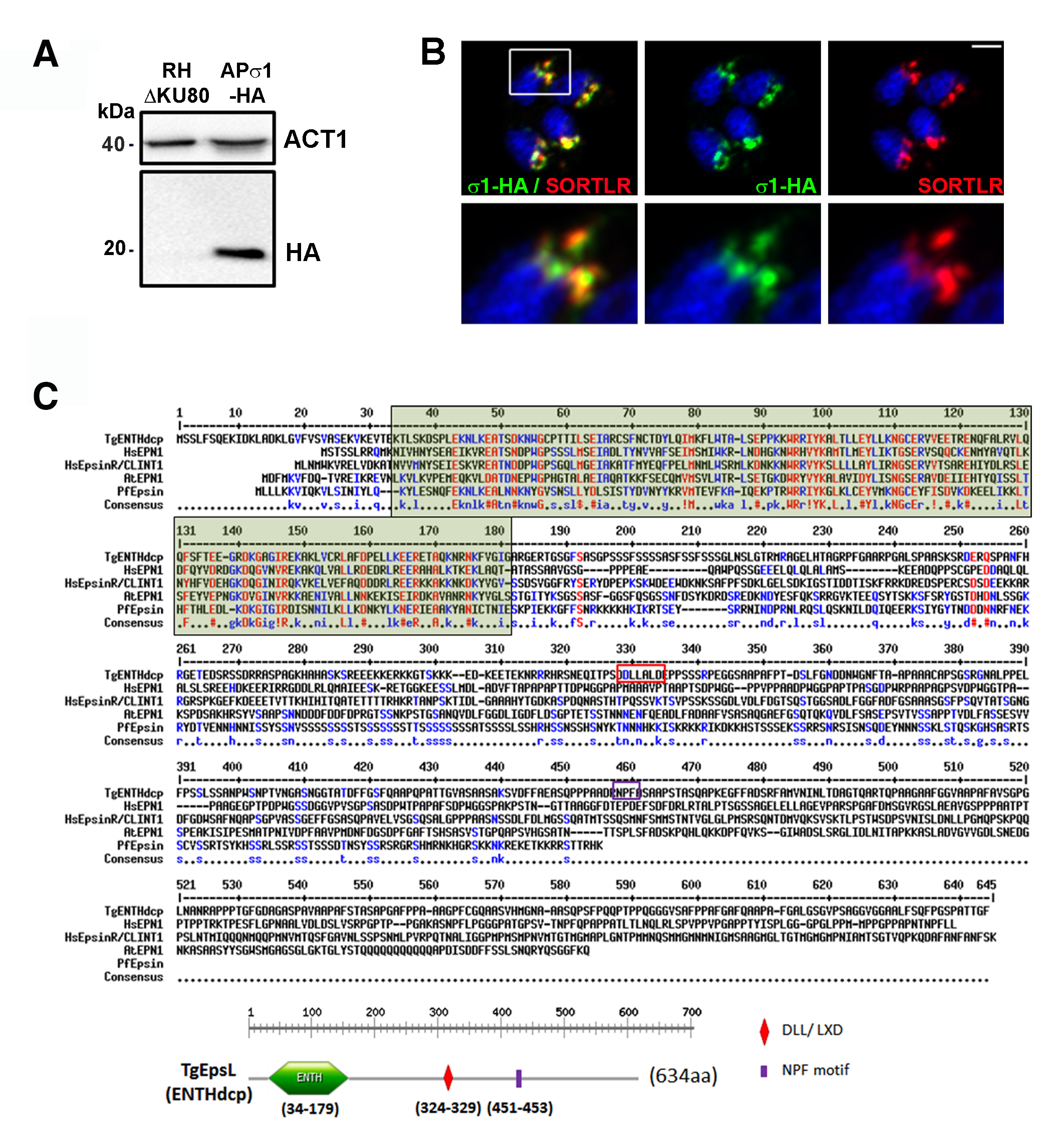

Supplement: S3 Fig — A- WB image showing the expression of the HA-tagged σ1 subunit of TgAP1 at the expected size (20 kDa) in KI parasites and its absence in the parental RHΔKU80 strain using anti-HA antibody. B- SIM images showing the localization of σ1-HA (green) at the TGN together with SORTLR (red). The white frame indicates the region zoomed in and shown as insets in the lower panel of images. Bar: 2 μm. C- Sequence alignment of the unique T. gondii ENTH-domain containing protein (TGGT1_214180) with Human epsin1 (Hs, UniProtKB: Q9Y6I3) and epsinR (CLINT1/Epsin4, UniProtKB: Q14677), with Arabidopsis thaliana epsinR2 (At, UniProtKB: Q67YI9) and Plasmodium falciparum (Pf) unique ENTH-domain containing protein (PF3D7_1245800). A scheme illustrating the positions of the identified conserved domains in TgEpsL, such as the ENTH domain, the clathrin binding site (DLL/LXD) and the NPF motif (the latter being predicted to mediate the association of epsin proteins with clathrin adaptor complexes), is shown below. (TIF) [file ppat.1006331.s003.tif]

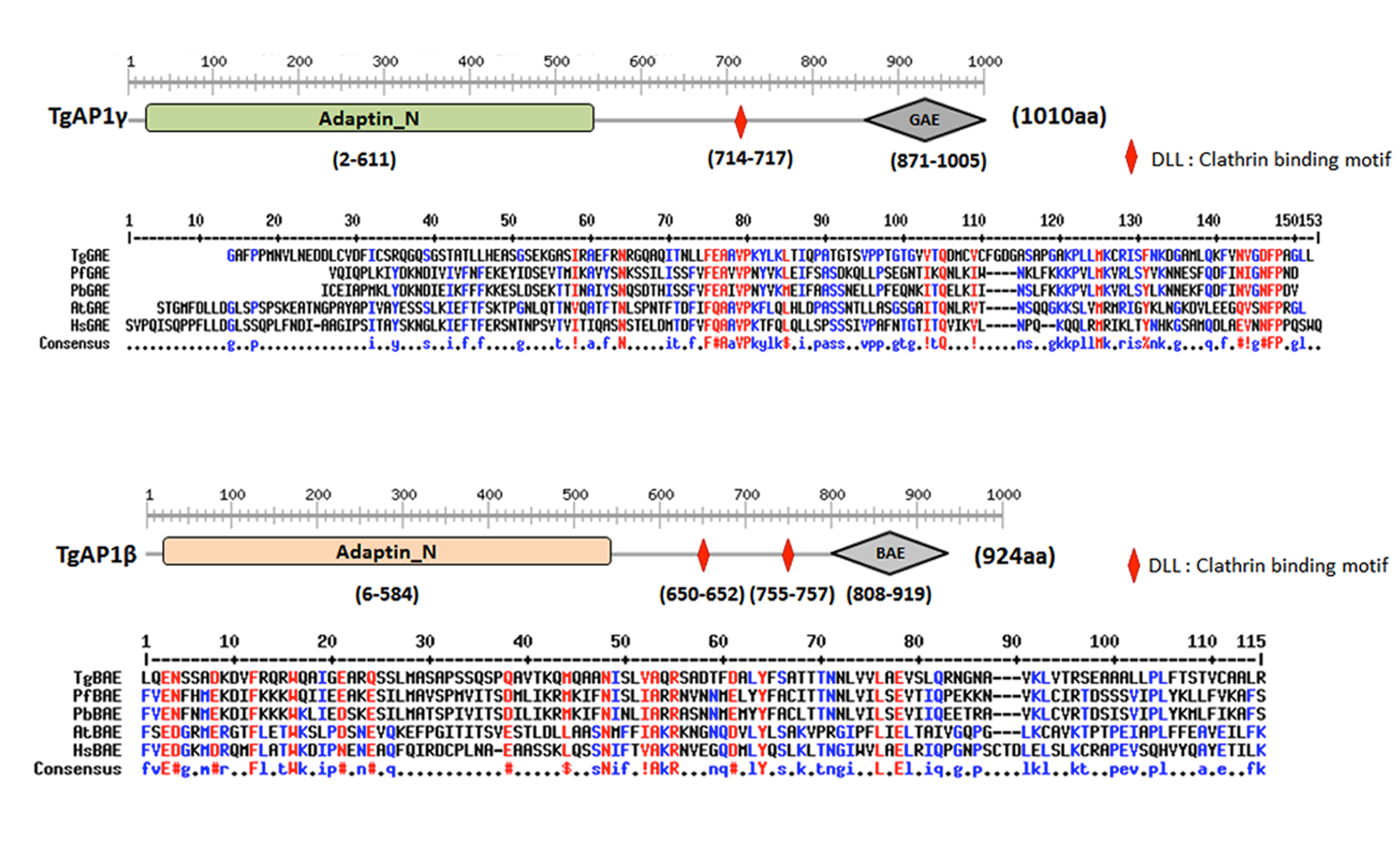

Supplement: S5 Fig — Sequence alignments of the GAE and BAE domains of T. gondii AP1 (Tg) with the corresponding sequences found in the AP1 complex of Plasmodium falciparum (Pf), P. berghei (Pb), Arabidopsis thaliana (At) and humans (Hs) are also shown. The accession numbers of analyzed proteins are indicated in the S1 Table. (TIF) [file ppat.1006331.s005.tif]

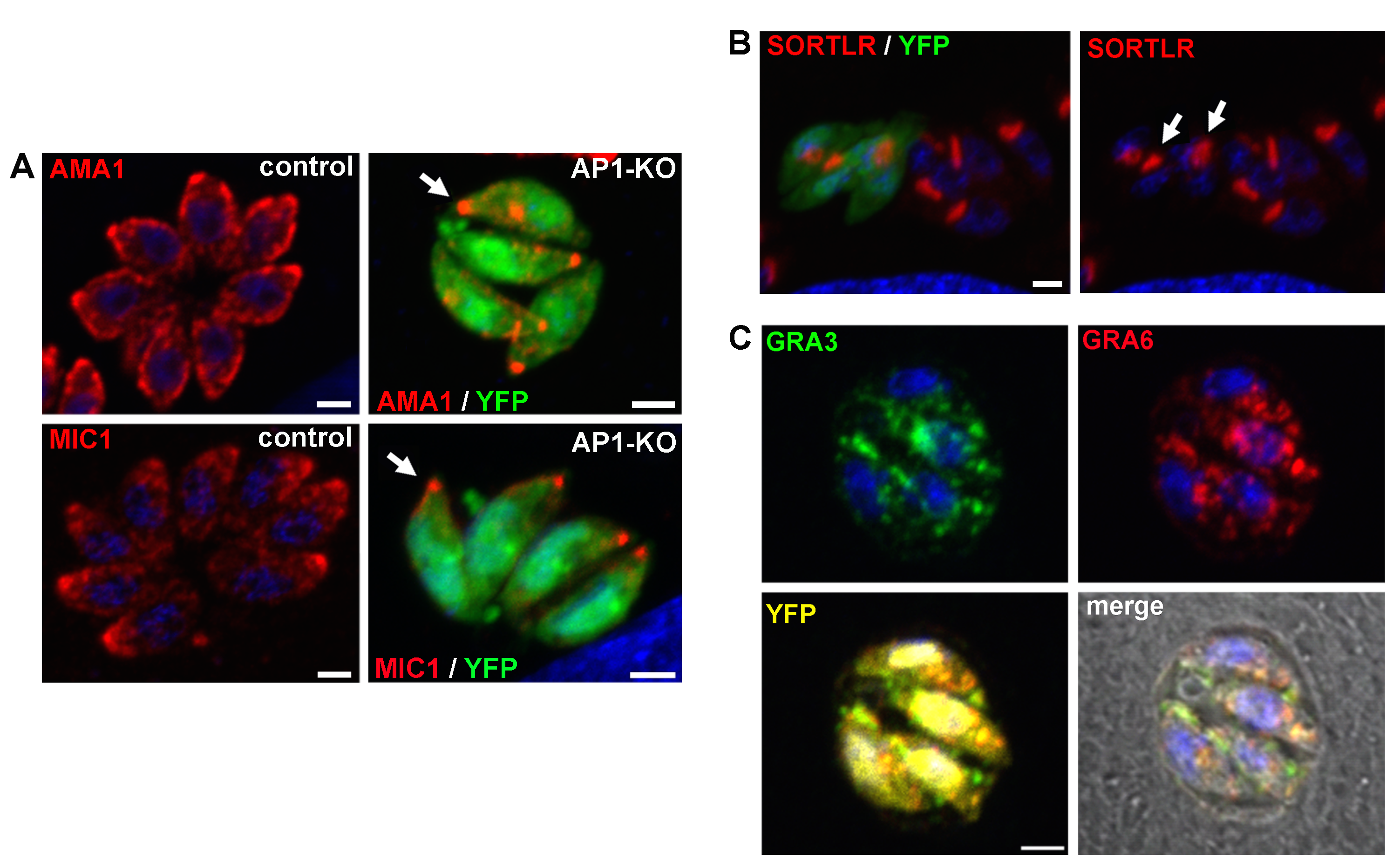

Supplement: S6 Fig — A, B- Confocal images showing the localization of AMA1 (A), MIC1 (A) and SORTLR (B) proteins (in red) in control (YFP-negative) and APμ1-KO parasites (YFP-positive). SORTLR was not found mis-localized in APμ1-KO. C- Confocal images showing the localization of GRA3 (green) and GRA6 (red) in YFP-positive APμ1-KO parasites (yellow). No defect in dense granule biogenesis was observed upon APμ1 depletion. All bars: 2 μm. (TIF) [file ppat.1006331.s006.tif]

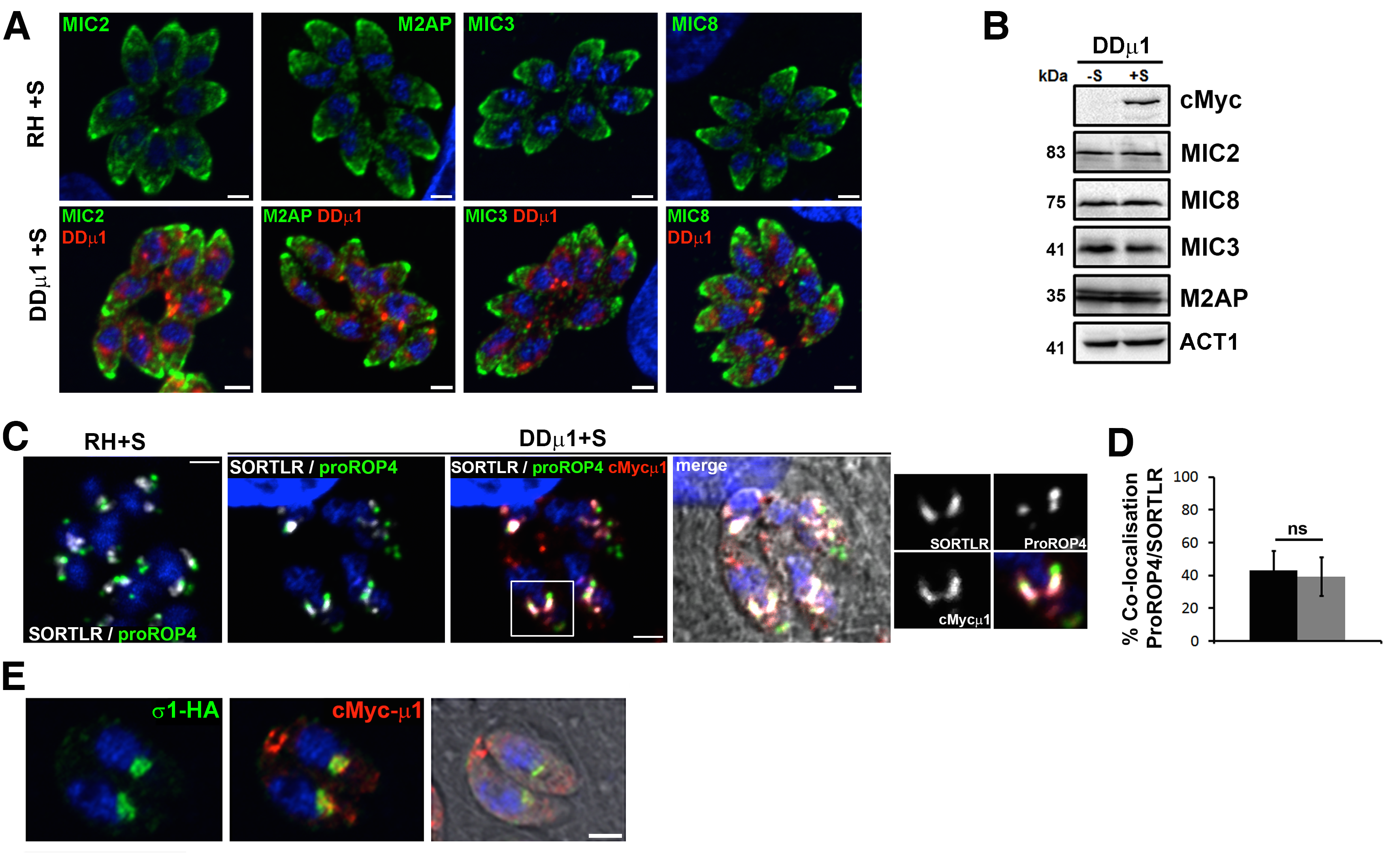

Supplement: S7 Fig — A- Confocal images showing the endogenous localization of MIC2, M2AP, MIC3 and MIC8 (all in green) in micronemes of DDμ1 parasites over-expressing the cMycμ1 protein (red) after shield-1 induction for 24 hours (+S). Bars: 2 μm. B- WB image showing no differences in the expression and proteolytic maturation of MIC2, MIC8, MIC3 and M2AP proteins in shield-1 induced control parasites (left) and DDμ1 parasites (right) for 24 h. Actin (ACT1) was used as loading control. C- Confocal images showing the co-localization of SORTLR (white) with proROP4 (green) and cMycμ1 (red) in control (RH) and DDμ1 parasites incubated with Shield-1 for 24 h. A zoom of the Golgi region indicated by a white frame on the merged image is shown as inset. Bars: 2 μm. D- Histogram indicating the percentage of co-localization between the proROP4 signal and the SORTLR signal after image acquisition by airyscan microscopy. Data are indicated as average ± SD, n≥15 vacuoles (Student’s t-test). E. Confocal images displaying the localization of the endogenous σ1-HA protein (green) in cMycμ1-overexpressing parasites (red) induced with shield-1 for 16 hours. The endogenous σ1-HA subunit was found localized at the Golgi together with the cMycμ1 protein. Bars: 2 μm. (TIF) [file ppat.1006331.s007.tif]

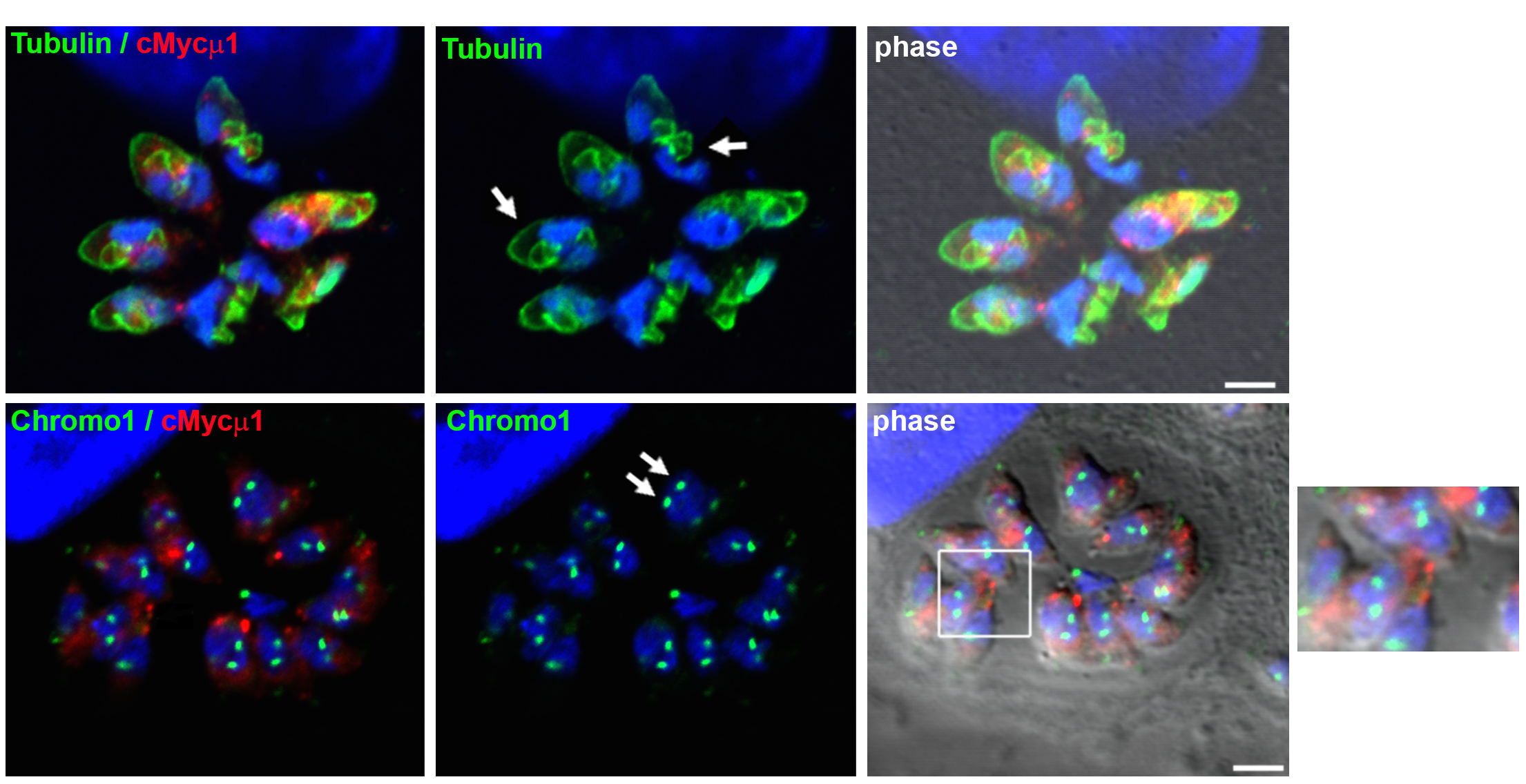

Supplement: S9 Fig — A zoom of the region indicated by a white frame in the merge image is shown in the inset on the right. Note the accumulation of the cMycμ1 protein at the basal pole of connected parasites. Bars: 2μm. (TIF) [file ppat.1006331.s009.tif]

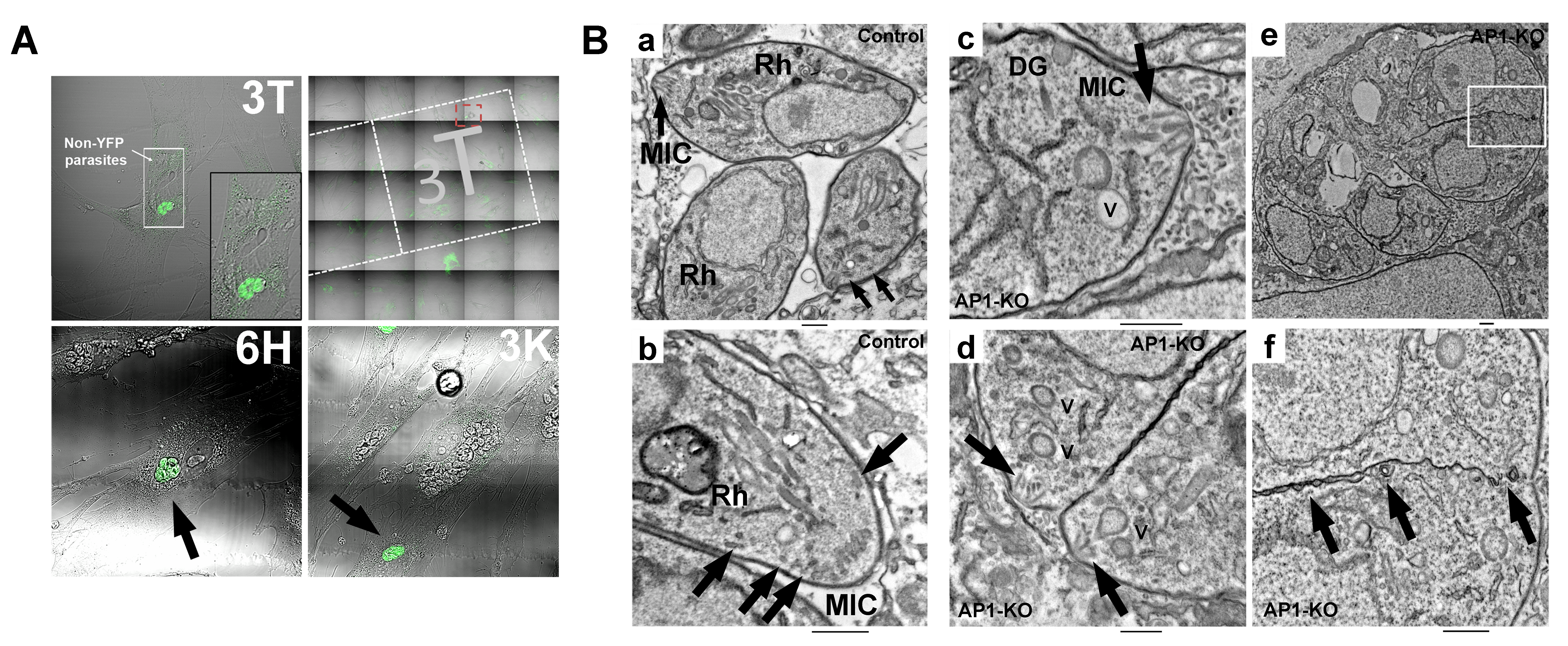

Supplement: S10 Fig — A- upper panel: strategy used to perform CLEM microscopy. HFF cells were allowed to grow (50% confluent) on alphanumerical coverslips. Confocal microscopy images were taken (40X objective) to spot the YFP positive parasites corresponding to APμ1-KO parasites or control non-YFP vacuoles. A mosaic of 8*8 microscopy fields centred on the parasites of interest was then acquired to determine the vacuole position on the grid as illustrated for the region 3T, corresponding to the APμ1-KO vacuole shown in Fig 9. The YFP-positive vacuoles at the positions 6H and 3K (arrows) correspond to vacuoles analyzed by CLEM and shown in B (image c: 6H and images d, e, f: 3K). B- Typical mature rhoptries and apical / lateral micronemes were detected in non-YFP control parasites (images a and b, arrows). By contrast, in YFP-positive APμ1-KO, no mature rhoptries were visualized at the apical pole, which instead displayed numerous big vesicles and only apical micronemes were detected (images c and d, arrows). Drastically affected APμ1-KO vacuoles showed abnormal division, with parasites that have broken out or not fully segregated (image e). Image f corresponds to a zoomed region of image e (white frame) showing incomplete pellicle formation and separation between two neighbouring cells (arrows). Rh: rhoptries, MIC: micronemes, DG: dense granules. Bars: 500nm. (TIF) [file ppat.1006331.s010.tif]

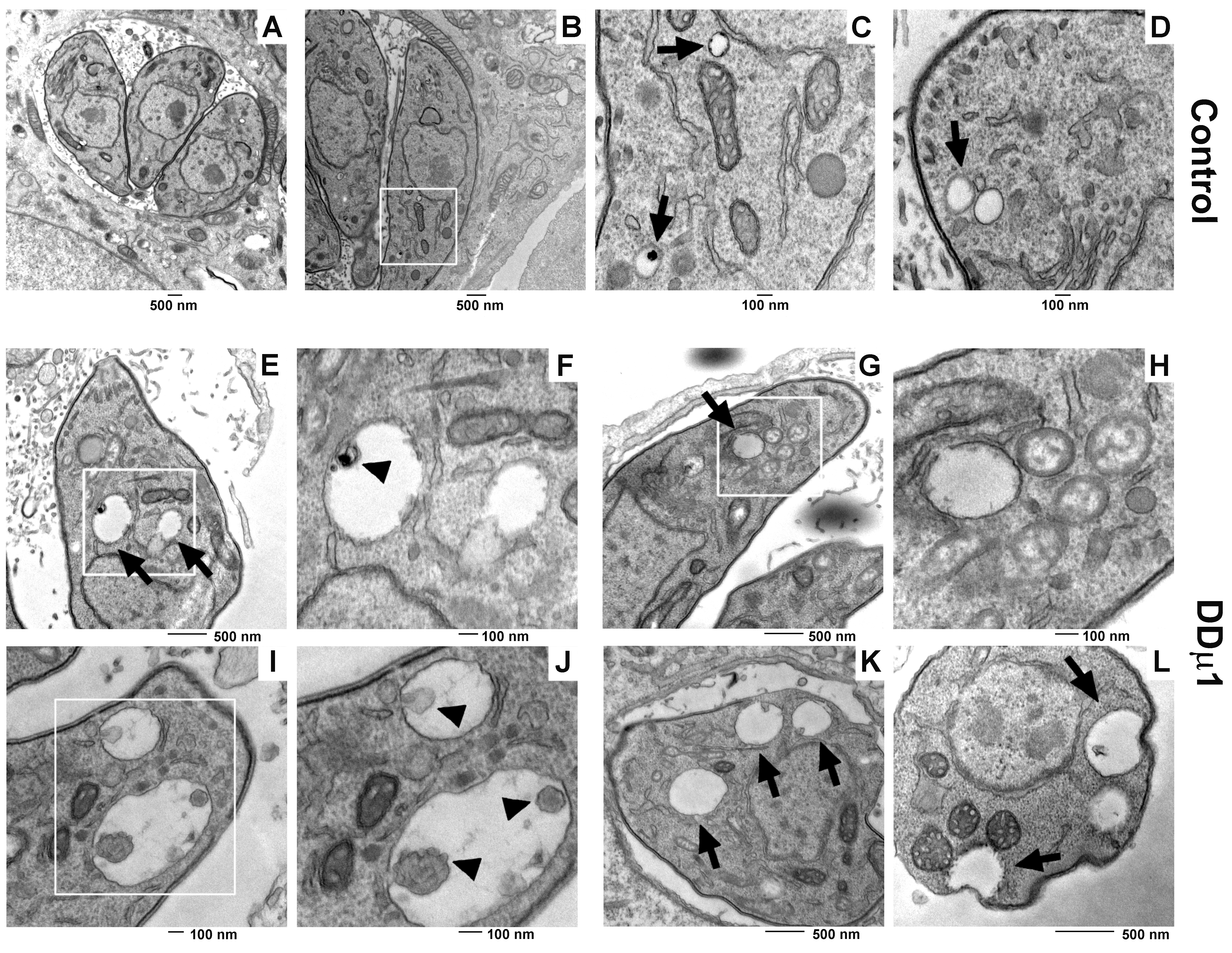

Supplement: S11 Fig — In control parasites (A-D), lucent vesicles were not often visualized or detected as small vesicles resembling endosomes (C and D, arrows). C represents a zoom of the region indicated by a white frame in B. In opposite, in induced DDμ1 parasites (E-L), large lucent vesicles accumulated mainly at the post-nuclear anterior region of the parasite (arrows). We often detected membranous or vesicle-like material within their lumen (E-F and I-J, arrow heads). In some cases, this material seems to be generated by internal budding of the limiting membrane (J, arrow heads). F, H and J represent a zoom of the region indicated by a white frame in E, G, and I, respectively. Bars: 100 nm or 500 nm as indicated. (TIF) [file ppat.1006331.s011.tif]
