# Supplementary material for: Dual role of the Toxoplasma gondii clathrin adaptor AP1 in the sorting of rhoptry and microneme proteins and in parasite division
Source: PLoS Pathog. 2017 Apr 21;13(4):e1006331. doi: 10.1371/journal.ppat.1006331 (PMC5415223; doi:10.1371/journal.ppat.1006331)
Supplement: S1 Table — (PDF) [file ppat.1006331.s012.pdf]

Table S1

**GAE domain alignment analysis**	**Accession number**
*Tg*AP1Gamma (*T. gondii*)	TGGT1_313670
*Pf*AP1Gamma (*P. falciparum*)	PF3D7_1455500
*Pb*AP1Gamma (*P. berghei* Anka)	PBANKA_1319200
*Hs*AP1Gamma (*Homo sapiens*)	UniProtKB - O43747
*At*AP1Gamma (*Arabidopsis thaliana*)	UniProtKB - Q84K16

**BAE domain alignment analysis**	**Accession number**
*Tg*AP1β *(T. gondii)*	TGGT1_240870
*Pf*AP1β *(P. falciparum)*	PF3D7_0528100
*Pb*AP1β *(P. berghei* Anka*)*	PBANKA_1242700
*Hs*AP1β *(Homo sapiens)*	UniProtKB - Q10567
*At*AP1β *(Arabidopsis thaliana)*	UniProtKB - O81742
